# Tobemstomig, a Novel Bispecific Antibody, Preferentially Blocks PD-1 and LAG-3 on CD8 TILs to Expand Stem-like T Cells for Sustained Tumor Control

**DOI:** 10.1158/2767-9764.CRC-26-0207

**Published:** 2026-07-09

**Authors:** Patrick A.A. Weber, Mario Perro, Valeria G. Nicolini, Klas Hatje, Silvia Jenni, Stefanie Briner, Esther Bommer, Manuela Tanner, Sylvain Pilet, Tamara Hüsser, Emilio Yángüez, Stanford Chen, Antonio Peixoto, Iryna Dekhtiarenko, Laura Lauener, Sylvia Herter, Thomas Hofer, Carina Hage, Meher Majety, Marina Bacac, Sara Colombetti, Stephane Leclair, Stefan Seeber, Merlind Mücke, Pablo Umaña, Christian Klein, Laura Codarri Deak

**Affiliations:** 1 Roche Innovation Center Zurich, Schlieren, Switzerland.; 2 Roche Innovation Center Basel, Basel, Switzerland.; 3 Pharmacology and Structural Biology, Toulouse University, CNRS, Toulouse, France.; 4 Roche Innovation Center Munich, Penzberg, Germany.; 5 Department of Biochemistry, Faculty of Chemistry and Pharmacy, Ludwig Maximilians University of Munich, Munich, Germany.

## Abstract

Checkpoint inhibitors targeting PD-1 have shown unprecedented efficacy in some patients with cancer; however, coexpression of the immune checkpoint LAG-3 by tumor-infiltrating lymphocytes (TILs) might hinder such efficacy. PD-1 and LAG-3 are established markers of T-cell exhaustion in cancer and are coexpressed by stem-like CD8 T cells, a population critical for the response to anti–PD-1 therapy, providing the rationale for their dual blockade. Yet, blocking LAG-3 can lead to the expansion of regulatory T cells (Treg), reducing the efficacy of anti–LAG-3 therapy on CD8 TILs. To address this limitation, we developed tobemstomig, a novel bispecific antibody (BsAb) to preferentially and simultaneously block PD-1 and LAG-3 in cis on tumor-specific CD8 TILs while sparing Tregs. It provided superior tumor growth inhibition in mouse models by replenishing stem-like cells and cytotoxic effector CD8 TILs, resulting in durable responses compared with monospecific antibodies targeting PD-1 and LAG-3 separately, which eroded the stem-like T-cell pool over time.

**Significance:**

Constitutive LAG-3 expression on Tregs may limit anti–LAG-3 therapy efficacy by promoting Treg expansion, leading to suppression of tumor-specific CD8 T-cell responses. Tobemstomig, a BsAb preferentially targeting CD8, expands tumor-specific stem-like CD8 TILs and reduces intratumoral Tregs, resulting in superior, durable tumor inhibition in mice.

## Introduction

Cancer immunotherapy (CIT) has provided unprecedented efficacy with the introduction of immune checkpoint inhibitors (CPI) targeting Programmed cell death protein 1 and Death-Ligand 1 (PD-(L)1) and Cytotoxic T-lymphocyte-associated protein 4 (CTLA-4) for the treatment of several malignancies (1–4). Nevertheless, many patients with cancer do not benefit from current CPIs because of primary or secondary resistance mechanisms, tumor-immune contexture, and regulatory T cells (Treg; ref. 5).

One potential reason for the development of resistance toward anti–PD-1 therapy is the coexpression of additional immune checkpoints with nonredundant regulatory functions like Lymphocyte-Activation Gene 3 (LAG-3) by tumor-specific T cells (6–13). PD-1 and LAG-3 are established markers of T-cell exhaustion (8, 14–18) in cancer and chronic viral infections; however, both receptors are also expressed by stem-like CD8 T cells (9, 19, 20), a population responsible for the efficacy of anti–PD-1 therapy (15, 19, 21, 22). PD-1 and LAG-3 prevent stem-like T-cell expansion, differentiation, and acquisition of effector functions (9, 11), thereby providing a solid rationale for therapeutically blocking both, PD-1 and LAG-3, pathways simultaneously.

There is indeed strong preclinical (11, 12, 17, 23–25) and clinical (13, 26–28) evidence supporting the benefit of coblocking PD-1 and LAG-3 over PD-1 alone. Nevertheless, an important limitation in achieving the full efficacy of LAG-3 blockade in cancer might be the constitutive expression of LAG-3 by Tregs. It has been shown that LAG-3 limits Treg proliferation and suppressive function (29). Therefore, blocking LAG-3 on Tregs might detrimentally increase their proliferation and suppressive function, potentially offsetting the benefit of blocking LAG-3 on tumor-specific CD8 tumor-infiltrating lymphocytes (TIL).

Here, we describe tobemstomig, a novel heterodimeric IgG1 bispecific antibody (BsAb) without Fc immune effector functions that preferentially blocks PD-1 and LAG-3 on tumor-specific CD8 TILs by targeting both receptors on the same T cell through an avidity-driven selectivity gain. The monovalent binding to LAG-3 together with the higher affinity monovalent binding to PD-1 combined with the different receptor expression levels determines tobemstomig’s preferential binding to CD8 TILs and conventional CD4 conventional T (Tconv) cells over Tregs. We have investigated tobemstomig’s properties, mechanism of action, and efficacy *in vitro* and *in vivo* in several mouse tumor models.

## Materials and Methods

### Generation of tobemstomig, a PD1–LAG3 bispecific, and its binding properties

Tobemstomig is a humanized PD1–LAG3 bispecific Fc-silent IgG1 antibody against the human PD-1 and the human LAG-3 receptors. It is engineered using CrossMab technology and charged variants for the correct assembly of the two different light chains, as well as “knob-into-hole” technology to promote the assembly of two different heavy chains (30). The variable regions of the PD-1 part of the bispecific were derived from immunizations in mice and the resulting hybridoma, followed by humanization, whereas the variable regions of the LAG-3 part of the PD1–LAG3 bispecific were derived from immunizations in transgenic rabbits, leading to a fully human anti–LAG-3 antibody. PD1–LAG3 bispecific is manufactured in a bioreactor using a suspension-adapted Chinese hamster ovary cell line.

### Human peripheral blood mononuclear cell isolation

The blood samples from healthy volunteers were obtained via the blood donation center (Zürich, Switzerland) with approval of the Cantonal Ethics Committee (Zürich). The peripheral blood mononuclear cells (PBMC) were isolated from the blood of different healthy donors using density gradient centrifugation with Histopaque-1077 (H8889-500M, Sigma-Aldrich). All cells were cultured in RPMI 1640 (11-875-135, Gibco) supplemented with 10% heat-inactivated FBS (F4135-500 mL, Sigma-Aldrich), GlutaMAX (35050061, Gibco), and 1% penicillin–streptomycin 100× (15140122, Gibco).

The PBMCs from 7 females, 12 males, and 5 unknown healthy donors born between 1944 and 1996 were used for *in vitro* experiments.

This study was conducted in accordance with the Declaration of Helsinki and was approved by the Swiss Ethics Committee (Business Administration System for Ethics Committees) under approval number 2020-01208 (valid until 2025). Written informed consent was obtained from all study participants prior to sample collection.

### Human CD4 or pan–T-cell isolation and *in vitro* activation

The human CD4 T cells were sorted using a CD4-positive selection Miltenyi beads system (CD4^+^ selection kit, 130-045-101, Miltenyi Biotec, RRID: AB_2889919) following the manufacturer’s instructions. Thereafter, the cells were labeled with carboxyfluorescein diacetate succinimidyl ester (CFSE; 5 μmol/L, 5 minutes at room temperature, 65-0850-84, eBioscience) or CellTrace Violet (CTV; 5 μmol/L, 5 minutes at room temperature, Thermo Fisher Scientific) to measure cell proliferation.

The human pan–T cells were sorted using a pan-negative selection bead system (Human Pan T Cell Isolation Kit, 130-096-535, Miltenyi Biotec, RRID: AB_3695577) following the manufacturer’s instructions.

The CD4 T cells or pan–T cells were seeded into an αCD3 precoated plate (1 μg/mL, clone OKT3, 317302, BioLegend, RRID: AB_571927, overnight, 4°C) with the addition of soluble αCD28 (1 μg/mL, clone CD28.2, 302902, BioLegend, RRID: AB_314304). The cells were cultured for 3 days to induce activation and upregulation of the PD-1 receptor on the surface of the T cells.

### Tobemstomig binding to activated CD4 T cells pretreated with competing anti–PD-1 or anti–LAG-3 antibodies

After 3 days of *in vitro* activation, the cells were harvested and washed several times. A portion of the activated T cells were exposed to 10 μg/mL of parental anti–PD-1 (P1AA6888, Roche) or anti–LAG-3 (P1AA1256, Roche) antibody to block the PD-1 or LAG-3 epitopes, respectively, for 30 minutes at room temperature, and thereafter the unbound antibody was washed away.

The cells were then resuspended in a 50 μL of PBS (10010031, Gibco) before the addition of 50 μL of a seven-step 10-fold dilution series of Alexa Fluor 647 (AF647)–directly labeled tobemstomig (PD1–LAG3–AF647, P1AE9562, Roche) from 66,000 to 0.066 pmol/L in PBS and incubated for 30 minutes at 4°C. After washing, the cells were stained at 4°C for CD4 (FITC, anti–human CD4, OKT4, 317408, BioLegend, RRID: AB_571951) and CD8 (BUV395 Mouse Anti-Human CD8, clone RPA-T8, 563795, BD Biosciences, RRID: AB_2722501) before fixation with BD CellFIX (554655, BD Biosciences) followed by acquisition with BD Biosciences Symphony A5 (RRID: SCR_022538) with FACSDiva (v9.1, BD Biosciences, RRID: SCR_001456) and analyzed using FlowJo software (FlowJo v10.8.1, BD Biosciences, RRID: SCR_008520). GraphPad Prism software (v8, GraphPad Prism, RRID: SCR_002798) was used for graphical representation and analyses.

### PD-1/PD-L1 and LAG-3/MHC-II Jurkat reporter assay

The PD-1/LAG-3 Jurkat reporter assay was performed following the manufacturer’s instructions. Briefly, the PD-1/LAG-3 Blockade Bioassay from Promega (PRJ-0103854) is a bioluminescent cell-based assay that overcomes the limitations of the existing primary cell-based assays and can be used to measure the potency of antibodies designed to block the PD-1/PD-L1 and LAG-3/MHC-II interactions.

The assay consists of two genetically engineered cell lines:

1. PD-1/LAG-3 effector cells: Jurkat T cells expressing a human PD-1 and human LAG-3 upon blockade lead to an individual downstream signaling and transcription of a luciferase reporter gene driven by an NFAT response element (NFAT-RE).
2. PD-L1/MHC-II antigen-presenting cells/A375 cells: A375 cells express human PD-L1 and MHC-II to be pulsed with a superantigen overnight before coculture with the Jurkat cell line to induce T-cell receptor (TCR) signaling.

When the two cell types are cocultured, the PD-1/PD-L1 and LAG-3/MHC-II interactions inhibit TCR signaling and NFAT-RE–mediated luminescence. The addition of either an anti–PD-1 or anti–LAG-3 antibody that blocks the PD-1/PD-L1 and LAG-3/MHC-II interactions releases the inhibitory signal and results in TCR activation and NFAT-RE–mediated luminescence.

Eight 10-fold dilutions from 100 μg/mL to 10 pg/mL of either in-house or competitor anti–PD-1 blocking antibodies (bivalent or monovalent anti–PD-1, nivolumab and pembrolizumab), parental bivalent or monovalent anti–LAG-3, or tobemstomig were added to the Jurkat cell line, right before coculturing them with the A375 cell line for 6 hours. After 6 hours of incubation at 37°C, we then added the substrate (G7941, Bio-Glo Reagent) before measuring the samples at the luminometer (Tecan Infinite M1000 Pro, RRID: SCR_025732). GraphPad Prism software (v8) was used for graphical representation and analyses.

### Internalization by activated CD4 T cells

#### Flow cytometry

Three days activated CD4 T cells, previously cultured with 1 μg/mL of plate-bound anti-CD3 and 1 μg/mL of soluble anti-CD28 antibodies, were incubated in duplicate in FACS tubes in the presence of parental anti–LAG-3, parental anti–PD-1, lead PD1–LAG3 BsAb (R07247669), or 2 + 1 or 2 + 2 PD1–LAG3 BsAb formats for 30 minutes at 4°C. The cells were then washed and divided into two groups, one of which was incubated for 3 additional hours at 37°C and the other one was immediately stained with a PE-labeled anti–human IgG secondary antibody (12-4998-82, eBioscience, RRID: AB_465926, 1:500) and anti-CD4 antibody (557871, BD Pharmingen, RRID: AB_396913, 1:50) before being fixed with BD CellFIX. After 3 hours of incubation, the second group of the cells was also stained with AF647-labeled anti-PGLALA (Roche) and APC-Cy7 anti-CD4 (clone RPA-T4, 1:50, 557871, BD Pharmingen) before fixation with CellFIX (BD Biosciences). The cells were then acquired at LSRFortessa (BD Biosciences, RRID: SCR_018655), and the expression levels of detectable antibody on the cell surface were compared between the two groups to calculate the percentage of LAG-3 internalization at 37°C using FlowJo software (FlowJo v10.8.1, BD Biosciences). GraphPad Prism software (v8) was used for graphical representation and analyses.

#### Confocal microscopy

Three days activated CD4^+^ T cells were stained with CellTracker Green 5-chloromethylfluorescein diacetate (CMFDA; C2925, Molecular Probes, Life Technologies) and plated on round coverslips treated with poly-L-lysine (A3890401, Sigma-Aldrich). The cells were allowed 30 minutes to adhere at 37°C before fluorescently tagged antibodies [1 μg/mL: anti–LAG-3, 1 + 1 (tobemstomig), 2 + 1, or 2 + 2 PD1–LAG3 BsAb antibody formats labeled with AF647] were added directly into the growth media for different durations (15 minutes, 1, 2, and 3 hours). Cold PBS (BEBP17-516Q, Lonza) was used to quench the reaction and to wash off unbounded antibodies. The cells were then fixed (51-2090KZ, BD Cytofix) for 20 minutes and washed twice with wash buffer (51-2090KZ, BD Cytoperm). After transferring the coverslips to a dry surface, they were then mounted on the glass slides with the mounting medium (00-4958-02, Fluoromount-G, eBioscience) and kept in the dark at 4°C before imaging. The intensity of the fluorescent signal from the membrane region of interest (ROI), of the targeted cells, was divided by the intensity of the fluorescent signal from the cytoplasm ROI of the same cells, resulting in a ratio displayed in the box charts. To compare the samples, one-way ANOVA analysis was used (*, *P* < 0.05; **, *P* < 0.001). Fluorescence confocal microscopy was performed with an inverted LSM 700 from Zeiss with a 60× oil objective. The images were collected using Zen software (Zeiss, RRID: SCR_013672) coupled to the microscope. The analysis of the images was performed with Imaris software (Bitplane; Oxford Instrument, RRID: SCR_007370), and the statistical analysis was performed using GraphPad Prism (GraphPad Software).

### Drug-shaving assay

#### Generation of human macrophages

The PBMCs were isolated from healthy donors and plated on Petri dishes. Upon monocyte attachment and clearing of the supernatant (including unattached/floating cells), the remaining monocytes were cultured at 37°C in a water-saturated atmosphere at 5% CO_2_. The cells were maintained using RPMI supplemented with 10% fetal calf serum (FCS), 1% GlutaMAX, 1% nonessential amino acids (11140035, Gibco), 5 μmol/L of β-mercaptoethanol (31350010, Gibco), and 1% antibiotic–antimycotic (15240062, Gibco). To foster their differentiation, the attached PBMCs were maintained in 50 to 60 ng/mL of human macrophage colony-stimulating factor (M-CSF; 300-25-10UG, Gibco) with medium changes every 3 days. On day 6, a new medium change was performed for 1 more days before measurements, in the absence of human M-CSF and in the presence of 20 ng/mL human IL-10 (200-10-10UG, PeproTech).

#### Isolation of CD8 T cells

The human CD8 T cells were isolated from donor-matched PBMCs using the Human CD8 T Cell Isolation Kit (130-096-495, Miltenyi Biotec, AB_3073903). The CD8 T cells were cultured on 24-well plates precoated overnight with an αCD3 precoated plate (1 μg/mL, clone OKT3, BioLegend, overnight, 4°C) with the addition of soluble αCD28 (1 μg/mL, clone CD28.2, BioLegend) to induce PD-1 expression. The cells were stimulated for 3 days before their use in the assay.

The macrophages were labeled with PKH in the plate just before data acquisition and prior to T-cell addition. The activated CD8 T cells were labeled with organic dyes (such as CMFDA), incubated with 1 μL/mL of AF647-labeled parental anti–PD-1, nivolumab, pembrolizumab, or tobemstomig, before coculturing them with autologous macrophages.

The time-lapse imaging was performed to visualize the shaving using confocal microscopy.

The acquired images were analyzed using Imaris software. The intensity of the drug on the T cells was extracted after cell segmentation and cell surface identification. The intensity data were analyzed using Excel and plotted in GraphPad Prism. A linear regression of each labeled drug intensity data in macrophages and T cells was calculated and displayed as a 95% prediction band. In addition, the intensity data were also normalized by the intensity of a control drug over time on T cells in the absence of macrophages (negative control of shaving) to provide the percentage of shaving in the 2-hour time frame.

### Binding competition on Treg and Tconv and Treg suppression assay

The CD4^+^ CD25^+^ CD127^dim^ Tregs were isolated from human peripheral blood with the two-step Regulatory T Cell Isolation Kit (130-091-301, Miltenyi Biotec). In parallel, the CD4^+^ CD25^−^ Tconv cells were isolated by collecting the negative fraction of a CD25^+^ selection (130-092-983, Miltenyi Biotec) followed by CD4^+^ enrichment (Miltenyi Biotec). Tconv cells were labeled with CFSE, and the Tregs were labeled with CTV to track the proliferation of both the populations.

For the PD-1, PD-L1, and LAG-3 receptor quantification and the binding competition, Tregs and Tconv cells were cocultured at a 1:1 ratio into a αCD3 precoated plate (1 μg/mL, clone OKT3, BioLegend) with soluble αCD28 (1 μg/mL, clone CD28.2, BioLegend). After 3 days of stimulation, a competitive binding assay was conducted with 1 μg/mL (6,600 pmol/L) of parental anti–PD-1, parental anti–LAG-3, atezolizumab, or tobemstomig, which were directly labeled with AF647. The directly coupled antibodies were incubated for 30 minutes at 4°C, and the cells were fixed with CellFIX (BD Biosciences).

The preferential antibody binding to Tconv cells or Tregs is calculated using the formula: Δ antibody binding = [mean fluorescence intensity (MFI) (Ab)Tconv − MFI (Ab)Treg] by subtracting the geometrical mean fluorescence of a specific antibody on Tregs from the geometrical mean fluorescence of Tconv cells. The positive values indicate a preferential binding to Tconv, the values around 0 indicate a similar binding to Tconv cells and Tregs, and the negative values would indicate a preferential binding to Tregs.

In the Treg suppression assay, the rescue of Tconv granzyme B production detected with AF647 anti–human granzyme B [clone GB11, 560212, BD Biosciences, RRID: AB_11154033, (1:100)] upon various treatments was measured upon coculturing Tconv cells together with Tregs at a 2:1 ratio for 5 days, in the presence or absence of treatment. The irradiated (40 Gy) feeders from an unrelated donor were used to elicit an allospecific stimulation. The suppression by Treg was calculated using the following formula:

$$\%\;\text{cytokine}\;\text{suppression}\;=\;100\;-\;\left(\frac{\;{\%\;\text{cytokine}}_{\left(\text{Tconv}+\text{Treg}\pm \text{blocking}\;\text{antibody}\right)}}{{\%\;\text{cytokine}}_{\left(\text{Tconv}\;\right)}}\;\times \;100\right)$$

where $\;{\%\;\text{cytokine}}_{\left(\text{Tconv}+\text{Treg}\pm \text{blocking}\;\text{antibody}\right)}$ is the level of cytokine secreted by Tconv cells in the presence of Tregs ± blocking antibody and ${\%\;\text{cytokine}}_{\left(\text{Tconv}\;\right)}$ is the level of cytokine secreted by Tconv cells in the absence of Tregs and without treatment.

### Flow cytometry staining for cytokine detection and receptor quantification

The cells were stained in PBS with surface antibodies for 30 minutes at 4°C and for being live/dead (either Aqua Dead Cell Stain, L34957, Invitrogen, during the last 10 minutes of incubation, or Fixable Viability Dye eFluor 780, 65-0865-14, eBioscience, for 30 minutes, 4°C). For intracellular staining, the cells were permeabilized with FACS permeabilization buffer (fixation/permeabilization, BD Biosciences; Foxp3 Transcription Factor Fixation Kit, 00-5523-00, eBioscience) and then incubated with antibodies specific for cytokines for 60 minutes at 4°C.

The numbers of PD-1, PD-L1, and LAG-3 receptors were quantified on the cell surface of human-activated Tregs and Tconv cells with the PE Phycoerythrin Fluorescence Quantitation Kit (340495, BD Biosciences) following the manufacturer’s instructions. Briefly, 2.5 μg/mL of PE-labeled monoclonal antibodies was used to quantify the receptor of interest on the gated populations of interest. The cells and the PE Quantibrite beads were fixed following the same protocol and fluorescence data acquired while using the same settings. The number of receptors was quantified following the kit instructions.

*The in vitro* binding of AF647–directly labeled tobemstomig, parental anti–PD-1, parental anti–LAG-3, or atezolizumab was performed via incubating 6,600 pmol/L of the constructs for 30 minutes on the activated Tregs and Tconv cells cultured together. After the washing step, the cells were incubated for an additional 30 minutes at 4°C with CD4 [clone OKT4 (1:100), BD Biosciences].

Sample acquisition was performed using BD Biosciences LSRIIFortessa or Symphony A5 with FACSDiva (version 9.1, BD Biosciences) and analyzed using FlowJo software (FlowJo v10.8.1-10.10, BD Biosciences). The graphs were generated using GraphPad Prism v8-10.3.1.

### Humanized mice

Female NOD.Cg-PrkdcscidIL2rgtm1Wjl/SzJ (NSG) mice (The Jackson Laboratory, RRID: IMSR_JAX:005557) and female NSG mice were bred by Charles River Laboratories. After arrival, the animals were maintained for 1 week to get accustomed to the new environment and for observation. Continuous health monitoring was carried out on a regular basis. The NSG mice were at the age of 4 to 5 weeks at the start of human stem-cell (HSC) engraftment, and they were maintained under specific pathogen–free condition with daily cycles of 12 hours of light/12 hours of darkness according to the committed guidelines (GV SOLAS, FELASA, and TierSchG). To generate HSC-engrafted NSG mice, the NSG mice were administered 15 mg/kg busulfan (L01AB01, Busilvex, Pierre Fabre) intraperitoneally. Twenty-four hours later, each mouse received an intravenous injection of 1 × 10^5^ human CD34^+^ cord blood cells (70008, STEMCELL Technologies). At 15 to 17 weeks after engraftment, the mice were screened, and only mice with more than 25% human CD45^+^ cells and a T-cell count greater than 80 cells/μL were enrolled in the studies. The efficiently engrafted mice were randomized according to their human T-cell frequencies into the different treatment groups.

For the BC004 patient-derived breast xenograft, female CD34^+^ humanized NOD.Cg-PrkdcscidIL2rgtm1Wjl/SzJ (NSG) mice (irradiation 140 cGy followed by intravenous injection of 9 × 10^4^ CD34^+^ cord blood cells/mouse) were obtained from The Jackson Laboratory. After reaching a human immune infiltrate (human CD45) above 25% in blood (14–16 weeks after humanization), the mice were shipped to Roche and maintained for 5 days to get accustomed to the new environment. The mice were kept under specific pathogen-free conditions with daily cycles of 12 hours of light/12 hours of darkness according to the committed guidelines (GV SOLAS, FELASA, and TierSchG). The experimental study protocol was reviewed and approved by the local government (ROB-55.2-2532.Vet_03-20-170).

### Transgenic mice

Sixty mixed-gender huPD-1xhuLAG-3 knock-in mice (genO-hPD-1/hLAG-3 mouse model, genOway) were maintained under specific pathogen–free conditions with daily cycles of 12 hours of light/12 hours of darkness according to the committed guidelines (GV SOLAS, FELASA, and TierSchG). The experimental study protocol was reviewed and approved by the local government (ZH225-17). After arrival, the animals were maintained for 1 week to become accustomed to the new environment and for observation. Continuous health monitoring was carried out on a regular basis.

### Mouse tumor models

#### WSU-DLCL2

The WSU-DLCL2 cells [human diffuse large B–cell lymphoma (DLBCL), RRID: CVCL_1902] were originally obtained from European Collection of Authenticated Cell Cultures (ECACC), and after expansion they were deposited in the Glycart internal cell bank. The cells were cultured in RPMI containing 10% FCS and 1× GlutaMAX. They were cultured at 37°C in a water-saturated atmosphere at 5% CO_2_. The *in vitro* passage P13 was used for subcutaneous injection with 1:1 GFR Matrigel (CLS356230, Corning), at a viability of 98.6%, using RPMI medium (w/o) in 100 μL, and 1.5 × 10^6^ cells were injected subcutaneously per mouse. Treatment began at day 14 when the tumor reached an average size of around 400 mm^3^.

Eighty-four fully humanized HSC-NSG mice were challenged subcutaneously with 1.5 × 10^6^ WSU-DLCL2 (human DLBCL, expressing CD20) at day 0 in the presence of Matrigel at a 1:1 ratio. At day 14 (tumor average around 350–400 mm^3^), a weekly scheduled therapy started: histidine buffer [vehicle (20 mmol/L of histidine and 140 mmol/L of NaCl, pH 6.0, 1000366, Bichsel)] as control; CD20-TCB (0.15 mg/kg once/week intravenously), CD20-TCB (0.15 mg/kg once/week intravenously) + nivolumab (1.5 mg/kg once/week intravenously), CD20-TCB (0.15 mg/kg once/week intravenously) + nivolumab (1.5 mg/kg once/week intravenously) + relatlimab-like anti–LAG-3 (1.5 mg/kg once/week intravenously), CD20-TCB (0.15 mg/kg once/week intravenously) + tobemstomig (1.5 mg/kg once/week intravenously), and CD20-TCB (0.15 mg/kg once/week intravenously) + anti–PD-1 (1.5 mg/kg once/week intravenously). All mice were injected intravenously of the appropriate solution. The mice in the vehicle group were injected with histidine buffer (1000366, Bichsel). To obtain the appropriate antibody concentration per 200 μL, the antibody solutions were diluted with histidine buffer when necessary.

The tumor volume was measured via a digital caliper 3 times a week. The tumor volume was measured over a period of 45 days.

The tumor volume was calculated with the formula:

Tumor volume=Length × Width × Depth × 4/3π

The animals were controlled daily for clinical symptoms and detection of adverse events. The tumor volume was measured via a caliper three times a week. The study exclusion criteria for the animals were described and approved in the corresponding license ZH193/2014.

The termination criteria for animals were visible sickness: scruffy fur, arched back, breathing problems, impaired locomotion, and tumor volume >3,000 mm^3^.

#### OCI-Ly18

The OCI-Ly18 cells (DLBCL, expressing CD20, RRID: CVCL_1880) were originally obtained from Deutsche Sammlung von Mikroorganismen und Zellkulturen GmbH (DSMZ), and after expansion they were deposited in the Glycart internal cell bank. The cells were cultivated in RPMI 1640 medium (42401-042, Gibco/LubioScience) containing 10% FCS (17593595, Gibco) and 1% GlutaMAX (35050-038, Invitrogen/Gibco).

The cells were cultured at 37°C in a water-saturated atmosphere at 5% CO_2_. The *in vitro* passage 14 was used for subcutaneous injection, at a viability of 98%, using RPMI medium (w/o). Briefly, 100 μL of the cell suspension containing 5 × 10^6^ OCI-Ly18 cells (1:1 RPMI:GFR Matrigel) was used for subcutaneous injection.

One hundred fully humanized HSC-NSG mice were challenged subcutaneously with 5 × 10^6^ OCI-Ly18 at day 0 in the presence of Matrigel at a 1:1 ratio. At day 11 (tumor average around 200 mm^3^), all the groups were treated with obinutuzumab for depletion of peripheral B cells. This was followed by a weekly scheduled therapy: histidine buffer as control; CD20-TCB (0.5 mg/kg once/week intravenously), CD20-TCB (0.5 mg/kg once/week intravenously) + tobemstomig BsAb (RO7247669, at 0.75, 1.5, or 3 mg/kg once/week intravenously), and CD20-TCB (0.5 mg/kg once/week intravenously) + pembrolizumab (1.5 mg/kg once/week intravenously) + favezelimab-like anti–LAG-3 antibody (1.5 mg/kg once/week intravenously).

All mice were injected intravenously of the appropriate solution. The mice in the vehicle group were injected with histidine buffer (1000366, Bichsel). To obtain the appropriate antibody concentration per 200 μL, the antibody solutions were diluted with histidine buffer when necessary.

The tumor volume was measured using a digital caliper 3 times a week. The tumor volume was measured over a period of 35 days. The study exclusion criteria for the animals were described and approved in the corresponding license ZH193/2014.

The tumor volume was calculated with the formula:

Tumor volume = Length × Width × Depth × 4/3π

#### BC004

The BC004 patient-derived xenograft (PDX), from breast expressing HLAG (Oncotest), was thawed as a single-cell suspension and prepared for *in vivo* injection in RPMI 1640 medium (31870025, Thermo Fisher Scientific) mixed with Matrigel (354230, Corning) at a 1:1 ratio. The BC004 PDX was injected into the intramammary fat pad of 70 fully humanized HSC-NSG mice at a concentration of 2 × 10^6^ cells per mouse at day 0. The mice were randomized in groups 1 day before treatment. The treatment began at day 35 when the tumor reached an average size of around 200 mm^3^. A weekly scheduled therapy started: phosphate–histidine buffer (vehicle) as control; HLAG-TCB (0.5 mg/kg once/week intravenously), HLAG-TCB (0.5 mg/kg once/week intravenously) + nivolumab (1.5 mg/kg once/week intraperitoneally) and relatlimab-like (1.5 mg/kg once/week intraperitoneally), HLAG-TCB (0.5 mg/kg once/week intravenously) + tobemstomig (1.5 mg/kg once/week intraperitoneally), and HLAG-TCB (0.5 mg/kg once/week intravenously) + tobemstomig (3 mg/kg once/week intraperitoneally).

The tumor growth was measured 2 to 3 times weekly using a caliper, and the tumor volume was calculated with the formula:

Tumor volume = Length × Width × Depth × 4/3π

The experimental study protocol was reviewed and approved by the local government (ROB-55.2-2532.Vet_03-20-170).

#### BxPC-3

The BxPC-3 cells (human pancreatic carcinoma cell line, expressing CEACAM5, RRID: CVCL_0186) were originally obtained from ECACC (ECACC 93120816), and after expansion they were deposited in the Glycart internal cell bank. The BxPC-3 cells were cultured in RPMI containing 10% FCS (PAA LABORATORIES) and 1% GlutaMAX. The cells were cultured at 37°C in a water-saturated atmosphere at 5% CO_2_. The *in vitro* passage 19 was used for subcutaneous injection, at a viability 98.8% using RPMI medium (w/o) and Matrigel 1:1 in a 100 μL final volume. The treatment began at day 15 when the tumor reached an average size of around 200 mm^3^.

Sixty-nine fully humanized HSC-NSG mice were challenged subcutaneously with 1 × 10^6^ BxPC-3 cells (human pancreatic carcinoma cell line, expressing CEACAM5) at day 0 in the presence of Matrigel at a 1:1 ratio. At day 19 (tumor average around 150–200 mm^3^), a weekly scheduled therapy started: PBS (vehicle) as control; CEACAM5 CD3 TCB (2.5 mg/kg once/week intravenously), CEACAM5 CD3 TCB (2.5 mg/kg once/week intravenously) + nivolumab (1.5 mg/kg once/week intraperitoneally), CEACAM5 CD3 TCB (2.5 mg/kg once/week intravenously) + nivolumab (1.5 mg/kg once/week intraperitoneally) + relatlimab-like anti–LAG-3 (1.5 mg/kg once/week intraperitoneally), CEACAM5 CD3 TCB (2.5 mg/kg once/week i.v.) + tobemstomig (3 mg/kg once/week intraperitoneally), and CEACAM5 CD3 TCB (2.5 mg/kg once/week intravenously) + anti–PD-1 (1.5 mg/kg once/week intraperitoneally). The tumor growth was measured 2 to 3 times weekly using a caliper, and the tumor volume was calculated with the formula:

Tumor volume = Length × Width × Depth × 4/3π

The animals were controlled daily for clinical symptoms and detection of adverse events. The tumor volume was measured using a caliper three times a week. The study exclusion criteria for the animals were described and approved in the corresponding license ZH193/2014.

The termination criteria for animals were visible sickness: scruffy fur, arched back, breathing problems, impaired locomotion, and tumor volume >3,000 mm^3^.

#### Panc02-H7-Fluc

The subcutaneous syngeneic models were used to assess the *in vivo* efficacy of tobemstomig compared with the single agents parental anti–PD-1, nivolumab, or the combination of nivolumab with relatlimab-like in C57BL/6J mice expressing the human PD-1 and LAG-3 extracellular domain of the receptors. Tumor growth inhibition (TGI) was the readout for the subcutaneous model. Briefly, 6- to 8-week-old female huPD-1 huLAG-3 tg C57BL/6J mice (Charles River Laboratories) were inoculated with 5 × 10^5^ Panc02-H7-Fluc (mouse pancreatic tumor, RRID: CVCL_D628) cells injected subcutaneously. The mice were maintained under specific pathogen–free conditions with daily cycles of 12 hours of light/12 hours of darkness according to the guidelines (temperature of 22°C, dark/light cycle of 12 hours, and humidity of 50%, GV SOLAS and FELASA), and food and water were provided *ad libitum*. Continuous health monitoring was carried out, and the experimental study protocol was reviewed and approved by the Veterinary Department of Canton Zurich.

The mice were randomized into different treatment groups and therapy when the tumors reached an average of 200 mm^3^ volume as measured using a caliper in the subcutaneous model. All treatments were administered intravenously, and the following doses were investigated: tobemstomig 10 mg/kg, parental anti–PD-1, and nivolumab and relatlimab-like at 5 mg/kg. The termination criterion for the orthotopic model for sacrificing animals was sickness with locomotion impairment, and the median overall survival was defined as the experimental day by which 50% of the animals had been sacrificed. The Kaplan–Meier survival curves and the pairwise log-rank test were used to compare survival between animal treatment groups. In the subcutaneous model, TGI was used as readout, and to test for significant differences in group means for multiple comparisons the standard ANOVA (one-way ANOVA) was used with the Dunnett method. The JMP statistic software program (SAS Institute, RRID: SCR_022199) was used for analyses.

Description of the subcutaneous model: The pancreatic carcinoma cell line Panc02-H7-Fluc (5 × 10^5^) was injected into the subcutis of the left flank. The tumor volume was measured using a caliper and calculated with the formula:

Tumor volume = Length × Width × Depth × 4/3π

The therapy was started when the tumor volume reached 150 to 200 mm^3^.

All the *in vivo* studies using mouse tumor models were approved by the Roche internal Institutional Animal Care and Use Committee, the animal licenses were approved by the Zurich cantonal veterinary authorities, and the experiments were performed in an Association for Assessment and Accreditation of Laboratory Animal Care International–accredited animal facility.

### Cell line authentication and contamination

The cell lines were authenticated through morphology and PCR assays with species-specific primers.

Fresh batches of the PD-1/LAG-3 Jurkat reporter cell line were regularly assessed for their expression profile of PD-1 and LAG-3.

The cell line batches were routinely tested for *Mycoplasma* and were negative.

### Study design

For the cancer model, *in vivo* efficacy studies sample size was determined using JMP statistic software (SAS Institute) to allow for significant difference with a minimum number of mice per group to comply with the country animal welfare guidelines. For mouse tumor models, randomization was performed using automated software in the POMES platform.

For *in vitro* studies, all used donors were either left untreated (negative control) or exposed in parallel to equimolar or binding site–matched concentrations of the indicated therapies.

For the mouse tumor models, the investigators were blinded to group allocation during data collection and analysis as treatment groups were replaced by alphabetical letters allocated by the people involved in preparing the drug dilutions for injections. The technical personnel were randomly assigned to studies during data collection and unaware of the hypothesis and expected outcome of the various treatments.

### Lymphocyte isolation

The mice were sacrificed according to the animal welfare guidelines; tumor tissue and blood were isolated in the animal facility. The tumor tissue was transferred into PBS and was disrupted using manual scissors and the Miltenyi gentleMACS machine (Miltenyi Biotec, RRID: SCR_020267). Subsequently, it was digested in an enzyme mix containing RPMI with 10 mg/mL DNase (11284932001, Sigma-Aldrich) and 0.25 mg/mL Liberase (05401020001, Sigma-Aldrich). Upon 30 minutes of digestion at 37°C, the tissue mix was filtered through a 70-μm strainer cell strainer (CLS431751, Corning) and resuspended to a single-cell suspension with an appropriate dilution for subsequent fluorescently labeled antibody staining. The blood was transferred in heparin tubes and was lysed with erythrocyte lysis buffer BD Pharm Lyse Lysing Buffer (555899, BD Biosciences). Upon red blood lysis, the cells were resuspended to a single-cell suspension with an appropriate dilution for subsequent fluorescently labeled antibody staining. The lymphocytes were mechanically isolated from the draining lymph nodes with a pestle, filtered through a 70-μm cell strainer, and resuspended as a single-cell suspension at an appropriate dilution for subsequent fluorescently labeled antibody staining.

### Reagents and flow cytometry

For the immunopharmacodynamic study on BxPC-3 tumors, the following antibodies were used: Fixable Viability Dye Aqua (1:500); V500 anti–human CD45 [clone HI30, 560779, BD Biosciences, RRID: AB_1937332 (1:50)]; AF700 anti–human CD3 [clone OKT3, 56-0037-42, eBioscience, RRID: AB_10714978 (1:50)]; APC-Cy7 anti–human CD4 (1:50); Pacific Blue anti–human CD8 [clone RPA-T8, BD Biosciences (1:50)]; either PE anti–human PD-1 [clone EH12.2H7, 329906, BioLegend, RRID: AB_940483 (1:20)], FITC anti–human LAG-3 [clone 17B4, LS-C344745-100, LSBio, RRID: AB_1650048, (1:20)], PE-Cy7 anti–human TIM-3 [F38-2E2, 25-3109-42, eBioscience, RRID: AB_2573438 (1:20)], AF647 anti–human granzyme B [clone GB11, 560212, BD Biosciences (1:100)], or PE anti–human Foxp3 [236A/E7, 12-4777-42, eBioscience, RRID: AB_1944444 (1:20)]; AF647 anti–human granzyme B (1:100); PE-Cy7 anti–human IFNg [4S.B3, 25-7319-82, eBioscience, RRID: AB_469682 (1:100)]; PerCP-eFluor710 anti-IL2 [MQ1-17H12, 46-7029-42, eBioscience, RRID: AB_1834419 (1:100)]; and FITC anti–human Ki67 [clone 20Raj1, 11-5699-42, eBioscience, RRID: AB_10687464 (1:100)].

In OCI-Ly18 and Panc02 tumors, to assess the *in vivo* binding of tobemstomig and *ex vivo* saturation, tumor single-cell suspension from vehicle and mice receiving two doses of tobemstomig were divided into two samples: one was left untreated, and the other one was saturated with 10 μg/mL of tobemstomig for 30 minutes at 37°C. After the washing step, the cells were stained with PE-conjugated anti-PGLALA antibody, which binds to the LALA-PG mutation in the Fc-portion of tobemstomig. For immunophenotyping, the following antibodies were used: PE-Cy7 antiFOXP3 human PD-1 [329918, BioLegend, RRID: AB_2159324 (1:100)], Fixable Viability Dye APC-Cy7 [L34975, Invitrogen (1:500)], BV421 anti–mouse FOXP3 [48-5773-82, eBioscience, RRID: AB_2716066, (1:100)], BV650 anti–mouse TIM-3 [119725, BioLegend, RRID: AB_2716066 (1:100)], BV785 anti–mouse CD3 [100232, BioLegend, RRID: AB_2562554 (1:100)], BUV395 anti–mouse CD8 [563786, BD Biosciences, RRID: AB_2732919 (1:100)], BUV495 anti–mouse CD4 [565974, BD Biosciences, RRID: AB_2739427 (1:100)], and BUV805 anti–mouse CD45 [612891, BD Biosciences, RRID: AB_2870179 (1:100)].

For immunopharmacodynamic studies in huPD-1, huLAG-3 double transgenic mice single-cell suspensions from tumors and blood were stained with the following antibodies listed: Fixable Viability Dye eFluor 455UV [65-0868-14, eBioscience, (1:500)], Alexa Fluor 700 CD45 [103128, BioLegend, RRID: AB_493715, (1:500)] or APC-Cy7 anti–mouse CD45 [clone 30-F11, 103116, BioLegend, RRID: AB_312981 (1:200)], BUV737 anti-TCRb [clone H57-597, 612821, BD Biosciences, RRID: AB_2870145 (1:500)], APC-Cy7 anti–mouse CD8 [clone 53-6.7, 100714, BioLegend, RRID: AB_312753 (1:500)] or PE-Cy7 anti–mouse CD8 [clone 53-6.7, 100722, BioLegend, RRID: AB_312761 (1:200)], BUV395 anti–mouse CD4 [clone GK1.5, 563790, BD Biosciences, RRID: AB_2738426 (1:500)], BV480 anti–mouse CD25 [566120, BD Biosciences, RRID: AB_2739522 (1:100)], BV605 anti–mouse TIM-3 [clone RMT3-23, 119721, BioLegend, RRID: AB_2616907, (1: 100)], PE anti–mouse TCF-1 [clone S33-966, 564217, BD Biosciences, RRID: AB_2687845 (1:200)], BV650 anti–human LAG-3 [clone 3DS223H, 416-2239-42, eBioscience, RRID: AB_2925692, (1:50)] or PE-Dazzle-594 anti–human LAG-3 [clone 3DS223H, 369218, BioLegend, RRID: AB_2894497 (1:20), Thermo Fisher Scientific], BV421 anti–mouse granzyme B [clone QA18A28, 396414, BioLegend, RRID: AB_2810603, (1:300)] or Alexa Fluor 700 anti–mouse granzyme B [clone QA18A28, 396426, BioLegend, RRID: AB_3741417 (1:100)], PE-Cy5 anti–mouse FoxP3 (clone FJK-16s, 15-5773-82, eBioscience, RRID: AB_468806) or BV421 anti–mouse FoxP3 [clone FJK-16s, 48-5773-82, eBioscience (1:100)], BV785 anti-Ki67 [clone SolA15 417-5698-82, eBioscience, RRID: AB_2925745 (1:300)], and Alexa Fluor 488 anti–mouse PD-1 [clone D7D5W, 34920, Cell Signaling Technology, RRID: AB_2799064 (1:200)], as the intracellular portion of PD-1 in the human PD-1 transgenic mice is murine.

For intracellular staining, the cells were fixed and permeabilized using the FOXP3 Transcription Factor Staining Buffer Set from eBioscience (00-5523-00, eBioscience) and CytoFix/CytoPerm Buffer or the Transcription Buffer Set from BD Biosciences.

For the detection of cytokines, tumor cell suspensions were restimulated with 6.25 ng/mL of phorbol 12-myristate 13-acetate (Sigma-Aldrich) and 1.87 μg/mL of ionomycin (Sigma-Aldrich) for 5 hours at 37°C. Upon 1 hour of restimulation, GolgiPlug (BD Biosciences) and GolgiStop (BD Biosciences) were added in the cell suspensions.

The discrimination of living cells versus dead cells was performed using 4′,6-diamidino-2-phenylindole (DAPI; Sigma-Aldrich) for the nonfixed samples and Fixable Viability Dye eFluor 780 (65-0865-14, eBioscience) for the fixed ones. The samples were acquired with a BD LSRIIFortessa and a BD FACSymphony A5 using FACSDiva (v9.1, BD Biosciences). Data obtained were analyzed using FlowJo software (v10.8.1, BD Biosciences).

### Cell sorting

The single-cell tumor suspensions were kept on ice during the staining and sorting procedure. The cell suspensions from three to five tumors of the same treatment group were stained with the following antibodies: Alexa Fluor 700 anti-CD45 [clone 30-F11, BioLegend (1:100)], BV711 anti-CD8 [clone 53-6.7, 100748, BioLegend, RRID: AB_2562100 (1:100)], bin channel BV605 anti-CD4 [clone GK1.5, 100451, BioLegend, RRID: AB_2564591 (1:100)], and BV605 anti-CD11c [clone N418, 117333, BioLegend, RRID: AB_11204262 (1:100)]. The discrimination of living cells versus dead cells was performed using Live/Dead APC-Cy7 [65-0865-14, eBioscience, (1:500)] for the nonfixed samples, and cells were incubated for 20 minutes. The cells were washed twice, filtered through a 40-μm cell strainer, and sorted on FACSAria III and acquired with FACSDiva (version 9.1, BD Biosciences) to enrich the viable, single, CD45^+^ CD8^+^ CD11c^−^ CD4^−^ population.

### Single-cell RNA isolation and RNA sequencing

The tumors were digested as previously described, and 1 to 10 Mio cells were stored in liquid nitrogen in the pZerve freezing medium (Z1653-20ML, Sigma-Aldrich). The samples were randomized and processed in four different batches with four samples each. After thawing a batch of samples, the cells were stained with a mix of FACS and oligo-labeled antibodies and sorted for CD3^+^ T cells before performing single-cell RNA sequencing (scRNA-seq). Briefly, the cells were washed once with PBS prior to the evaluation of both the cell number and viability using a Nexcelom Cellometer Auto 2000. Approximately 1 Mio cells per sample were resuspended in 50 μL of PBS and incubated with 5 μL of Mouse TruStain FcX Fc Blocking reagent (101319, BioLegend, RRID: AB 1574973). The mix of FACS and oligo-labeled antibodies was added to the cells in a volume of 50 μL (final volume 100 μL). After 30 minutes of incubation at 4°C, the cells were washed 3 times with PBS and resuspended in 500 μL of PBS + 1% BSA to get a concentration of approximately 1 × 10^6^ cells/mL. The cells were filtered through a 40-μm cell strainer and sorted in a BD FACSAria II system. The cell number and viability of the sorted cells were determined using a Nexcelom Cellometer Auto 2000, and a total of 10,000 viable cells per sample were loaded into the 10x Genomics Chromium Connect Instrument. cDNA and library preparation were performed according to the manufacturer’s indications (10x Genomics Chromium Next GEM Automated Single Cell 5′ Reagent Kits version 2 with TCR and feature barcoding), and the resulting libraries were sequenced in an Illumina NovaSeq 6000 sequencer according to 10x Genomics recommendations (R1 = 26; i7 = 10; i5 = 10; and R2 = 90) to a depth of approximately 20,000 reads per cell for the GEX library and 5,000 reads per cell for both the TCR and feature barcoding libraries.

### scRNA-seq analysis of TILs

The FASTQ files were generated using 10x Genomics Cell Ranger (8.0.1mkfastq, 10x Genomics, RRID: SCR_023897). To estimate unique molecular identifier (UMI) counts and gene expression levels, the reads were mapped to the murine genome (mm39) utilizing 10x Genomics Cell Ranger 8.0.1 count. The resulting raw gene-by-cell count matrix per sample was filtered for empty droplets using Dropkick (31). Filtered per-sample count matrices were merged, and to achieve a high-quality dataset, low-quality cells with a low amount of genes, low UMI count depth, or a high fraction of mitochondrial reads were removed using thresholds of at least 1,000 and not more than 40,000 UMIs per cell and thresholds derived by the median absolute deviation method with a factor of 3 deviations, resulting in at least 827 genes and not more than 1.41% of UMIs mapping to mitochondrial genes. Normalization was performed using count depth scaling to 10,000 total counts per cell, resulting in the cp10k (counts per 10,000) unit. The count values were log-transformed using natural logarithm: ln (cp10k + 1).

The filtered and normalized gene-by-cell count matrix was further processed using Scanpy (RRID: SCR_018139; ref. 32). To obtain a clustering for cell-type annotation, we selected the top 2,000 highly deviant genes, scaled the matrix to a mean of 0 and variance of 1, calculated the first 50 principal components, identified the 10 nearest neighbors per cell, and applied the Leiden clustering. We annotated cell types using cell-type signatures provided by Besca (33). The signature enrichment scores were calculated by the average expression of the signature genes after subtraction by the average expression of a randomly sampled reference set of genes (scanpy.tl.score_genes).

### Statistical analysis

Prism software (version 8-10.3.1, GraphPad Prism) was used for statistical analysis. A one-way or two-way analysis of variance (ANOVA) test was used for comparing more than two groups. To test for significant differences in TGI between group means for multiple comparisons, the standard ANOVA (one-way ANOVA) was used with the Tukey *post hoc* test.

## Results

### Generation of tobemstomig, a PD1–LAG3 BsAb

**Figure 1.**
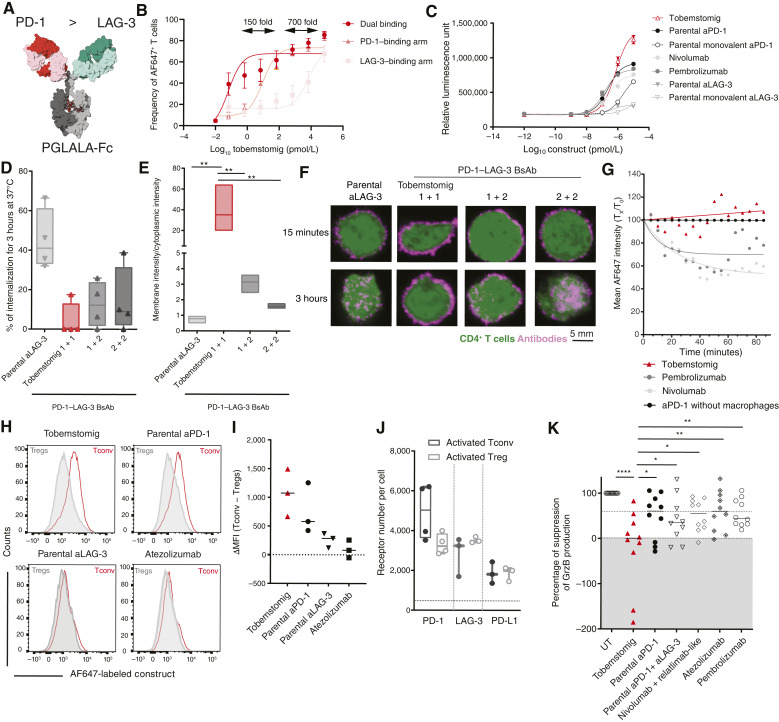
Tobemstomig is not internalized or shaved by macrophages and simultaneously binds/blocks PD-1 and LAG-3 on the same cell resulting in preferential targeting of Tconv cells over Tregs rescuing them from Treg suppression. **A,** Tobemstomig structure. **B,** Frequency of *in vitro* activated, polyclonal human AF647^+^ CD4 T cells upon exposure for 30 minutes to increasing concentrations of AF647-labeled tobemstomig. Part of the PD-1^+^ LAG-3^+^ T cells were pretreated with competing PD-1 antibody or LAG-3 antibody to prevent PD-1– or LAG-3–mediated targeting of tobemstomig (*n* = 4 healthy donors, two independent experiments; mean ± SEM). **C,** Relative luminescence unit of PD-1 and LAG-3 Jurkat reporter cell line upon exposure to PD-1 and LAG-3 blocking antibodies (*n* = 3, three independent experiments; mean ± SEM). aLAG-3, anti–LAG-3; aPD-1, anti–PD-1. **D,** Percentage of internalized construct by polyclonally activated CD4 T cells after 3 hours of incubation at 37°C detected via flow cytometry (*n* = 4 healthy donors, four independent experiments; box plots represent median, minimum/maximum, and individual points). **E,** Ratio of construct on cell membrane vs. cytoplasm of polyclonally activated CD4 T cells measured via confocal microscopy. The intensity of the fluorescent signal from the membrane ROI of targeted cells was divided by the intensity of the fluorescent signal from the cytoplasm ROI of the same cells (*n* = 3 healthy donors, three independent experiments; box plots represent median and minimum/maximum). **F,** Representative confocal images depicting assessed constructs on single polyclonally activated CD4 T cells after 15 minutes and 3 hours of incubation. T cells labeled with CMFDA (green) during incubation with labeled antibodies (purple). One-way ANOVA with uncorrected Fisher's least significant difference (LSD) (*, *P* < 0.05; **, *P* < 0.01). **G,** Intensity signal of the fluorescent antibodies from Imaris plotted over time. The dataset was normalized over control (parental anti–PD-1) to define a percentage of loss of signal from the T cells defined as the percentage of shaving. A linear regression was then calculated based on normalized data. **H,** FACS histogram plots of binding competition of directly conjugated tobemstomig, parental PD-1 antibody, LAG-3 antibody, or atezolizumab to human CD4 Tconv cells vs. Tregs, cultured together, from one representative donor of three. **I,** ΔMFI of human CD4 Tconv cells and Tregs stained with either labeled tobemstomig, parental PD-1 antibody, parental LAG-3 antibody, or atezolizumab (*n* = 3 healthy donors, median). **J,** Amount of PD-1, LAG-3, and PD-L1 receptors per T cell on Tconv cells and Tregs (*n* = 4 healthy donors; box plots represent median, minimum/maximum, and individual points). **K,** Treg suppression of Tconv granzyme B (GrzB) secretion in the presence of 66,000 pmol/L of the indicated treatments (*n* = 10 healthy donors, five independent experiments; median). Two-way ANOVA with the Tukey multiple comparison test (*, *P* < 0.05; **, *P* < 0.01; ****, *P* < 0.0001). UT, untreated.

Tobemstomig is a humanized Fc-silenced IgG1 BsAb with a 1 + 1 format that binds with a monovalent high-affinity arm to the human PD-1 receptor (250 pmol/L at 37°C – 500 pmol/L at 25°C) and with a monovalent lower affinity arm to the human LAG-3 receptor (780 pmol/L at 37°C – 11.1 nmol/L at 25°C; Fig. 1A). Chain mispairing is prevented by the introduction of knob-into-hole mutations and the use of CrossMab technology (30). On PD-1, it recognizes a unique glycopeptide epitope that largely overlaps with the binding sites of nivolumab and pembrolizumab, thereby interfering with PD-L1 and PD-L2 interactions. The LAG-3–binding arm recognizes a novel epitope (E3) that is different from relatlimab and fianlimab’s epitope E5 within the domain D1. The difference in affinity between the PD-1– and LAG-3–binding arms is intended to achieve an avidity-driven selectivity binding of tumor-specific T cells that coexpress both receptors on their surface, in particular stem-like T cells. Tobemstomig does not interact with Fcγ receptors (FcγR) because of the introduction of P329G LALA mutations into the human IgG1 Fc portion, which prevents antibody-dependent cellular cytotoxicity, antibody-dependent cellular phagocytosis, and complement-dependent cytotoxicity while retaining neonatal Fc receptor binding enabling IgG-like pharmacokinetics (34).

### Relative contributions of tobemstomig-binding arms to PD-1 and LAG-3

To assess the relative contribution of tobemstomig-binding arms to PD-1 and LAG-3, polyclonally activated CD4 T cells were either left untreated or preincubated with a competing antibody toward PD-1 or LAG-3, before being exposed to increasing concentrations of tobemstomig directly labeled with AF647. We observed that the half maximal effective concentration (EC_50_) of the bound tobemstomig on untreated cells was 0.06 pmol/L because of the binding avidity of the PD-1 and LAG-3 binders when binding simultaneously in cis (Fig. 1B; Table 1). However, on T cells pretreated with a competing antibody targeting LAG-3, tobemstomig relied solely on the binding of the PD-1 arm leading to an increase in EC_50_ to 9.3 pmol/L, indicating a roughly 150-fold loss in potency (Fig. 1B; Table 1). Interestingly, when we pretreated the T cells with a competing antibody targeting PD-1 to assess the contribution of the LAG-3-binding arm, the EC_50_ concentration further increased to roughly 6,500 pmol/L corresponding to a 10^5^-fold loss in potency when compared with untreated cells. This indicates a roughly 700-fold difference in potency between the PD-1- and the LAG-3-binding arms (Fig. 1B; Table 1).

**Table 1.** Binding contributions of the PD-1 and LAG-3 arms of tobemstomig.

Binding potency	Untreated	aLAG-3 pretreated	aPD-1 pretreated
EC_50 _(pmol/L)	0.06	9.30	6,496
Fold increase in EC_50_ from untreated	1	146.71	102,379.82

Abbreviations: aLAG-3, anti–LAG-3; aPD-1, anti–PD-1.

To confirm that the observed binding behavior of tobemstomig was driven by the relative binding affinities for PD-1 and LAG-3, and resulting avidity gain, rather than a consequence of the different expression densities of the two receptors, we quantified the PD-1 and LAG-3 receptors on the activated CD4 T cells with the PE Phycoerythrin Fluorescence Quantitation Kit. We found that both PD-1 and LAG-3 were expressed at the same densities as the number of receptors per T cells (Supplementary Fig. S1A). This confirms that the binding behavior of tobemstomig is indeed the result of the relative affinities of the individual binders and the resulting avidity gain when both receptors are bound in cis on the same cell.

### Tobemstomig blocks the PD-1/PD-L1 and LAG-3/MHC-II pathways simultaneously

Subsequently, in a cellular assay, we measured the synergistic activity of coblocking the PD-1/PD-L1 and LAG-3/MHC-II pathways with tobemstomig using a PD-1 and LAG-3 Jurkat reporter cell line. This cell line has been genetically engineered to express human PD-1 and LAG-3 receptors both modulating the luciferase expression through an NFAT-RE. Upon incubation with an artificial antigen-presenting cell line expressing human PD-L1 and MHC-II in the presence of a superantigen, the TCR signaling and the NFAT-RE mediate the luminescence of the PD-1/LAG-3 Jurkat reporter cell line. However, the respective interactions of PD-1 with PD-L1 and LAG-3 with MHC-II inhibit the TCR signaling and the NFAT-RE–mediated luminescence, unless blocking antibodies are used to prevent those interactions, resulting in a partial or total TCR stimulation and luminescence.

In this assay, tobemstomig was approximately threefold less potent (EC_50_) than the molecular weight-matched parental bivalent anti–PD-1 antibody and 1.8-fold less potent than the parental bivalent anti–LAG-3 antibody (Fig. 1C; Table 2). However, tobemstomig was threefold more potent than the monovalent anti–PD-1 and sixfold more potent than the monovalent anti–LAG-3 (Fig. 1C; Table 2). In addition, the maximal effect (E_max_) induced by tobemstomig was roughly 30% or higher than the one of all the bivalent anti–PD-1 antibodies and more than 70% higher than the one of all the bivalent anti–LAG-3 antibodies. The higher E_max_ of tobemstomig also translated into a higher area under the curve than the other treatments (Fig. 1C; Table 2).

**Table 2.** Dose–response curves of PD-1 and LAG-3 blockade in the PD-1/LAG-3 Jurkat reporter cell line.

​Potency/efficacy	Pembrolizumab	Nivolumab	Bivalent parental aPD-1	Monovalent parental aPD-1	Monovalent parental aLAG-3	Bivalent parental aLAG-3	Tobemstomig
EC_50 _(pmol/L)	1.38E−07	2.57E−07	2.31E−07	2.13E−06	4.35E−06	3.85E−07	7.07E−07
C_max_	847,404.5	763,271.5	914,930	659,229	331,735	303,236.5	1,266,968
AUC	7.832	6.551	8.19	4.899	2.647	3.013	10.6
EC_50_ fold-decrease (higher potency) vs. tobemstomig	5.13	2.75	3.06	0.33	0.16	1.84	1
C_max_ fold-decrease vs. tobemstomig	1.50	1.66	1.38	1.92	3.82	4.18	1
% C_max_ decrease relative to tobemstomig	33.12	39.76	27.79	47.97	73.82	76.07	0
ΔAUC from tobemstomig	2.768	4.049	2.41	5.701	7.953	7.587	0

Abbreviations: AUC, area under the curve; C_max_, maximal (or peak) concentration; aLAG-3, anti–LAG-3; aPD-1, anti–PD-1.

Due to its monovalency, despite blocking only half of the PD-1 and LAG-3 receptors than the monospecific bivalent antibodies at the same concentrations, tobemstomig showed to have only a slight reduction in potency while having a much higher maximal effect due to the synergism derived from the simultaneous blockade of the PD-1 and LAG-3 pathways.

### Tobemstomig is less internalized by T cells

It has been previously described that LAG-3 is internalized through endocytosis upon interaction with its ligand α-syn (35), although LAG-3 internalization following binding to the other ligands, like MHC-II, galectin-3, and FGL-1, has not been reported. Considering that half of the LAG-3 receptors are stored intracellularly, the endocytosis of the LAG-3 receptors once bound to an antagonist antibody could promote the quick translocation of free LAG-3 from the cytoplasm to the T-cell surface (36), representing a significant sink for therapeutic antibodies aimed to block LAG-3. Therefore, we assessed the internalization of LAG-3 upon binding to antagonistic antibodies in different formats.

For this purpose, polyclonally activated CD4 T cells were exposed in duplicates to a subsaturating concentration of either bivalent parental anti–PD-1, anti–LAG-3, or PD1–LAG3 BsAbs in different formats including tobemstomig. After 30 minutes or 3 hours of incubation, the samples per treatment group were immediately stained with an AF647-labeled anti-PGLALA antibody, which specifically binds to the P329G mutation present on the primary antibodies, before fixation (20). All the cells were then acquired via flow cytometry, and for each treatment the levels of detectable fluorescent antibody on the cell surface of the samples incubated at 37°C were compared with the one at 4°C. We observed that after 3 hours at 37°C, roughly 45% of the parental bivalent anti–LAG-3 antibody was internalized by the T cells, whereas only 12.5% and 14%, respectively, of the 1 + 2 (monovalent for PD-1 and bivalent for LAG-3) and 2 + 2 formats of PD1–LAG3 BsAbs were internalized (Fig. 1D). Conversely, we observed less than 5% internalization for tobemstomig (Fig. 1D).

To corroborate these findings, we further assessed the internalization via confocal microscopy. This time polyclonally activated CD4 T cells plated on the coverslips were exposed to a subsaturating concentration of AF647–directly labeled antibodies at 37°C for 15 minutes or 3 hours before fixation. Tobemstomig showed a roughly 40-fold higher signal intensity from the cellular membrane than from the cytoplasm, which was significantly higher than the other bispecific formats, with only three- and twofold, respectively, and the parental anti–LAG-3 antibody (Fig. 1E and F).

These data altogether show that tobemstomig has a longer retention on the surface of T cells probably due to its high-affinity monovalent binding to PD-1, which works as an anchor. This suggests a reduced sink effect and, therefore, a potentially improved bioavailability for tobemstomig over other bispecific formats and bivalent anti–LAG-3 antibodies.

### Tobemstomig is refractory to drug shaving by macrophages

Another important sink which has been described for IgG1- and IgG4-based anti–PD-1 therapies is the tumor-associated macrophage–mediated drug shaving (37). Although the anti–PD-1 antibodies bind to PD-1^+^ CD8 TILs after administration, they are quickly removed through the Fc:FcγR interaction and degraded by tissue-resident macrophages (37).

To assess whether tobemstomig lacking FcγR binding is subjected to drug shaving by macrophages, we generated macrophages from monocytes isolated from healthy donor PBMCs and cocultured them with polyclonally activated CD8 T cells pretreated with directly conjugated parental, competitor anti–PD-1 antibodies, or tobemstomig.

To visualize the shaving process, we ran time-lapse imaging over 80 minutes for all the compounds. Within the experimental timeframe, we observed a 40% and 25% decrease in the amount of nivolumab and pembrolizumab present on the surface of T cells, respectively (Fig. 1G; Supplementary Fig. S1B–S1D). Interestingly, due to the PGLALA mutation in the Fc-portion, tobemstomig was not removed and degraded by macrophages and, therefore, remained on the surface of T cells over time (Fig. 1G; Supplementary Fig. S1B–S1D).

This observation suggests that tobemstomig is refractory to drug-shaving by tissue and tumor-resident macrophages, which represent an additional sink for IgG1- and IgG4-based therapies like current PD-1 and LAG-3 CPIs. This might result in a higher receptor occupancy by tobemstomig on T cells in the tumor resulting in an improved tumor retention and a higher tumor exposure.

### *In vitro* tobemstomig preferentially binds to Tconv cells and rescues them from Treg suppressive function

Tregs represent a limitation for the success of several CITs (20), and a recent research has shown that LAG-3 on Tregs seems to negatively regulate their suppressive function (29). Therefore, the use of LAG-3 blocking antibodies, which indiscriminately target LAG-3 on Tregs and effector T cells, could detrimentally increase the suppressive function of Treg eventually offsetting the benefit of blocking LAG-3 on tumor-reactive T cells.

Therefore, we performed a binding competition assay, in which Tconv cells and naturally occurring Tregs from human peripheral blood were labeled with two different membrane dyes, before being cultured together in equal number in the presence of polyclonal stimulation. Three days later, the cells were exposed to a nonsaturating concentration of either AF647–directly conjugated parental anti–PD-1, anti–LAG-3, atezolizumab, or tobemstomig followed by the assessment of their binding behavior via flow cytometry. Although the parental anti–LAG-3 antibody and atezolizumab bound equally to Tregs and Tconv cells (Fig. 1H), the parental anti–PD-1 and tobemstomig preferentially bound to Tconv cells than to Tregs (Fig. 1H). This preferential binding to Tconv cells can also be visualized as the difference in the geometric mean fluorescent intensity signal from Tconv cells and the one from Tregs (Fig. 1I). Consistent with this finding, when we quantified the target receptor densities on Tconv cells and Tregs, we found approximately a twofold higher number of PD-1 receptors per T cell on Tconv cells than on Tregs, although LAG-3 and PD-L1 receptor densities were comparable on the two T-cell subsets (Fig. 1J).

To assess whether the preferential binding of tobemstomig to Tconv cells would provide them with a functional advantage over Tregs, we performed a suppressive function assay. Tconv cells and Tregs were cultured together for 5 days in the presence of allogeneic PBMCs obtained from an unrelated donor to provide discrete TCR stimuli. We measured the ability of Tregs to dampen Tconv effector functions such as granzyme B secretion, which in the absence of treatment was normalized to 100% to reduce interdonor variability. The addition of an anti–PD-1, parental or pembrolizumab, reduced the suppression to 50% to 60% (Fig. 1K). Interestingly, the combination of anti–PD-1 with anti–LAG-3 antibodies, either parental or an analog to relatlimab, only minimally decreased the suppression of granzyme B by Tregs (Fig. 1K). In contrast, tobemstomig significantly overcame Treg suppression and rescued Tconv effector functions when compared with the other treatments (Fig. 1K).

These data suggest that the preferential binding of tobemstomig to Tconv cells is beneficial for eliciting their effector functions due to a reduced detrimental activation of Tregs via LAG-3 targeting.

### Tobemstomig provides better TGI and eradication than the combination of anti–PD-1 and anti–LAG-3 antibodies

**Figure 2.**
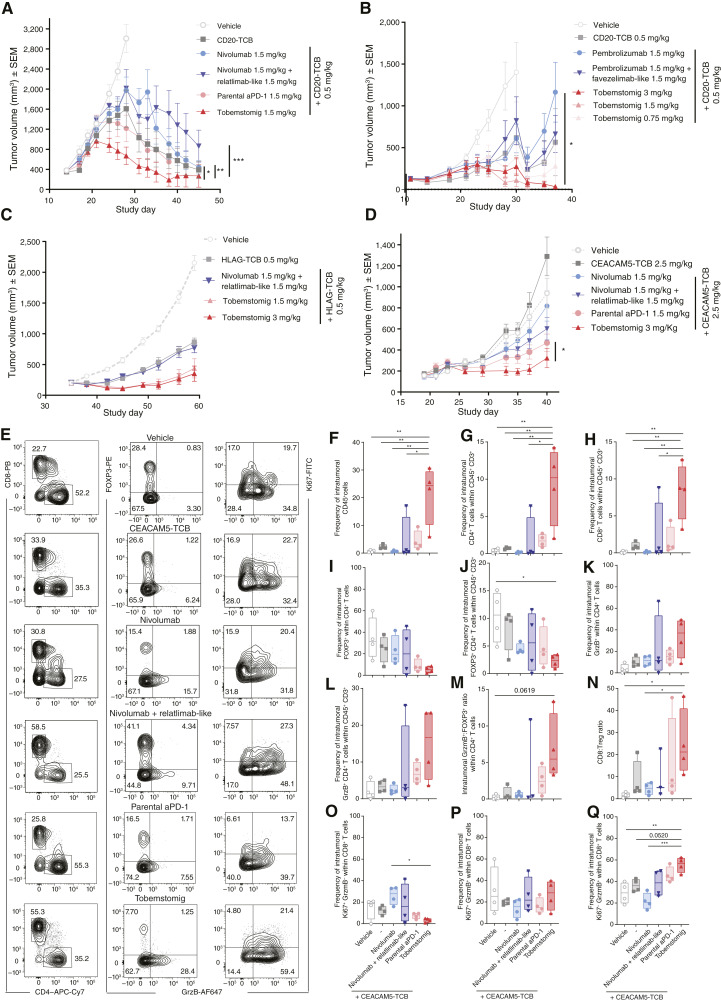
Tobemstomig provides TGI and eradication in several mouse tumor models by favoring the expansion of cytotoxic CD4 and CD8 TILs over Tregs. *In vivo* efficacy studies in CD34^+^ humanized NSG mice bearing subcutaneous or intramammary fat pad tumors treated for 4 weeks with the indicated treatments. **A,** Tumor growth curves of WSU subcutaneous tumors in humanized control mice and mice treated with the indicated therapies (*n* = 10 mice per treatment group, mean ± SEM). aPD-1, anti–PD-1. **B,** Tumor growth curves of OCI-Ly18 subcutaneous tumors in humanized control mice and mice treated with the indicated therapies (*n* = 14 mice per treatment group, mean ± SEM). **C,** Tumor growth curves of BC004 intramammary fat pad tumors in humanized control mice and mice treated with the indicated therapies (*n* = 14 mice per treatment group, mean ± SEM). **D,** Tumor growth curves of BxPC-3 subcutaneous tumors in humanized control mice and mice treated with the indicated therapies (*n* = 10 mice per treatment group, mean ± SEM). **E,** Representative contour plot depicting the effect of different treatments on CD4 and CD8 frequencies (gated on CD3^+^ T cells), Tregs (FOXP3^+^) vs. cytotoxic (granzyme B^+^) CD4 TILs (gated on CD4^+^ T cells), and proliferating (Ki67^+^) vs. cytotoxic (granzyme B^+^) CD8 TILs (gated on CD8^+^ T cells) isolated from BxPC-3 tumors. **F–Q,** Immunopharmacodynamic study depicting the effect of different treatments on CD45^+^ cells, CD4 and CD8 frequencies (gated on CD45^+^CD3^+^ T cells), Tregs (FOXP3^+^) vs. cytotoxic [granzyme B^+^ (GrzB^+^)] CD4 TILs, and proliferating (Ki67^+^) vs. cytotoxic (granzyme B^+^) CD8 TILs, expressed either as frequencies within the CD4^+^ or CD8^+^ T-cell gate, respectively (**I**, **K**, and **O–Q**), as frequencies within CD45^+^CD3^+^ T cells (**J** and **L**), or as intratumoral ratios (**M** and **N**), isolated from BxPC-3 tumors. The Kruskal–Wallis with Dunn *post hoc* test was used to compare TGI across treatment groups in the various mouse tumor models. The Mann–Whitney U test was used to compare TGI between tobemstomig and parental anti–PD-1. One-way ANOVA with the Tukey multiple comparison test was used to compare treatment effects on TIL subsets (*, *P* < 0.05; **, *P* < 0.01; ***, *P* < 0.001).

To better characterize the differentiating features of tobemstomig, we next performed *in vivo* efficacy studies in mice. In the absence of a surrogate BsAb binding with comparable relative affinities with murine PD-1 and LAG-3 receptors, therefore recapitulating tobemstomig features and properties, we performed the efficacy studies in humanized mice. Due to an undeveloped myeloid compartment, however, the T cells of these mice are not properly primed and therefore unable to mount an endogenous adaptive immune response toward the tumor, requiring the use of a T-cell BsAb to provide signal-1 (38). For this reason, humanized mice bearing a B-cell lymphoma (WSU) were treated once a week for 4 weeks with CD20-TCB as monotherapy or in combination with either tobemstomig or nivolumab with relatlimab-like anti–LAG-3. Parental anti–PD-1 and nivolumab in combination with CD20-TCB were used as the reference to estimate the relative contribution of LAG-3 blockade by tobemstomig and relatlimab-like combination, respectively. CD20-TCB as monotherapy led to TGI and tumor rejection within 45 days from tumor challenge (Fig. 2A).

A Mann–Whitney U test comparison of TGI between tobemstomig and parental anti–PD-1 revealed a significant contribution of the LAG-3 blockade to tobemstomig efficacy (*P* = 0.0042), whereas it showed lack of statistical difference between nivolumab monotherapy and its combination with relatlimab-like (*P* = 0.5). Interestingly, the combination of CD20-TCB with tobemstomig induced a significantly better control of the tumor growth and led to an even faster tumor rejection than CD20-TCB in combination with nivolumab (*P* = 0.006; Fig. 2A). Surprisingly, the combination of nivolumab with relatlimab-like anti–LAG-3, despite blocking twice the amount of the PD-1 and LAG-3 receptors, did not recapitulate the efficacy observed for tobemstomig (*P* = 0.0002; Fig. 2A).

We next assessed three different doses of tobemstomig in CD20-TCB–resistant B-cell lymphoma model (OCI-Ly18) and compared its efficacy with pembrolizumab as monotherapy and in combination with favezelimab-like anti–LAG-3. In this mouse tumor model, CD20-TCB could control the tumor growth for some weeks, but ultimately the tumor escaped (Fig. 2B). The blockade of the PD-1 pathway with pembrolizumab did not provide any additional benefit to CD20-TCB treatment (Fig. 2B). Importantly, tobemstomig showed a dose–efficacy relationship, providing tumor growth control and eradication with doses equal and higher than 1.5 mg/kg, and both the doses were significantly more efficacious than pembrolizumab (*P* < 0.03; Fig. 2B). Conversely, the combination of pembrolizumab with favezelimab-like anti–LAG-3 was not significantly better in providing TGI than pembrolizumab alone. Therefore, also in this case, the combination of monospecific anti–LAG-3 and anti–PD-1 antibodies did not recapitulate the efficacy observed for tobemstomig (Fig. 2B). After two *in vivo* administrations of tobemstomig, some mice were sacrificed to assess its *in vivo* binding behavior to TILs. Notably, tobemstomig showed a dose-dependent preferential binding to CD8 TILs over Tconv cells and Tregs across all three doses (Supplementary Fig. S2A and S2B). At 3 mg/kg, tobemstomig reached 100% receptor occupancy on CD8 TILs and Tconv cells, and roughly 70% on Tregs (Supplementary Fig. S2C), supporting the concept of a preferential binding toward CD8 and conventional CD4 TILs over Tregs.

In a mouse model of patient-derived tumor explant (BC004) of breast cancer, nivolumab in combination with relatlimab-like anti–LAG-3 did not provide additional benefit to mice treated with HLAG-TCB, whereas tobemstomig showed a trend of improved efficacy (*P* = 0.059; Fig. 2C). The highest dose of tobemstomig, tested to match the number of blocked receptors, showed a trend for having a better TGI than nivolumab in combination with relatlimab-like (*P* = 0.07; Fig. 2C).

Finally, in mice bearing a pancreatic adenocarcinoma cell line (BxPC-3) treated with CEACAM5-TCB, tobemstomig showed a trend toward an improved tumor growth control over CEACAM5-TCB alone (*P* = 0.07; Fig. 2D). The Mann–Whitney U test comparison of TGI between tobemstomig and parental anti–PD-1 revealed, once more, a significant contribution of the LAG-3 coblockade, in addition to PD-1, to tobemstomig efficacy (*P* = 0.018; Fig. 2D). Conversely, the same type of comparison between nivolumab in combination with relatlimab-like versus nivolumab monotherapy failed to show a significant difference (*P* = 0.86). Immunopharmacodynamic analysis of the BxPC-3 tumors at termination revealed an increase in frequencies of tumor-infiltrating immune cells upon tobemstomig treatment (Fig. 2E and F). Both the frequencies of CD4 and CD8 TILs significantly increased upon tobemstomig therapy, when compared with the vehicle group and other treatment groups (Fig. 2G and H). A further characterization of the CD4 T-cell functional phenotype revealed a significant decrease in the frequencies of Tregs (Fig. 2E, I, and J) accompanied by an increase in cytotoxic Granzyme B^+^ CD4 TILs (Fig. 2E, K, and L), resulting in a higher ratio of intratumoral cytotoxic CD4 T cells to Tregs (Fig. 2M). Similarly, tobemstomig significantly favored a higher CD8 TIL:Treg ratio (Fig. 2N) and significantly decreased the frequency of proliferating Ki67^+^ CD8 TILs (Fig. 2E, O, and P) while increasing those of cytotoxic Granzyme B^+^ CD8 TILs (Fig. 2E, K, and Q).

Altogether, these data support an improved and differentiated efficacy profile for tobemstomig which is not recapitulated by the combination of anti–PD-1 and anti–LAG-3 antibodies. *In vivo*, tobemstomig preferentially targeted CD8 and conventional CD4 TILs over Tregs and increased the frequencies of cytotoxic CD8 and CD4 TILs while decreasing those of Tregs, consistent with its preferential binding to CD8 and conventional CD4 TILs over Tregs as observed *in vitro* and *in vivo*.

### *In vivo* tobemstomig preferentially binds and expands CD8 TILs including PD-1^+^ TCF-1^+^ stem-like T cells and drives their differentiation toward cytotoxic effectors

To address whether indeed the preferential targeting of tobemstomig to CD8 TILs was responsible for the observed *in vivo* efficacy, we performed an efficacy study in immunocompetent human PD-1 and human LAG-3 double transgenic mice bearing a subcutaneous syngeneic pancreatic adenocarcinoma (Panc02-H7-Fluc). These mice allow to better investigate the mechanism of action of tobemstomig in the absence of a T-cell BsAb which might affect the phenotype and function of T cells.

**Figure 3.**
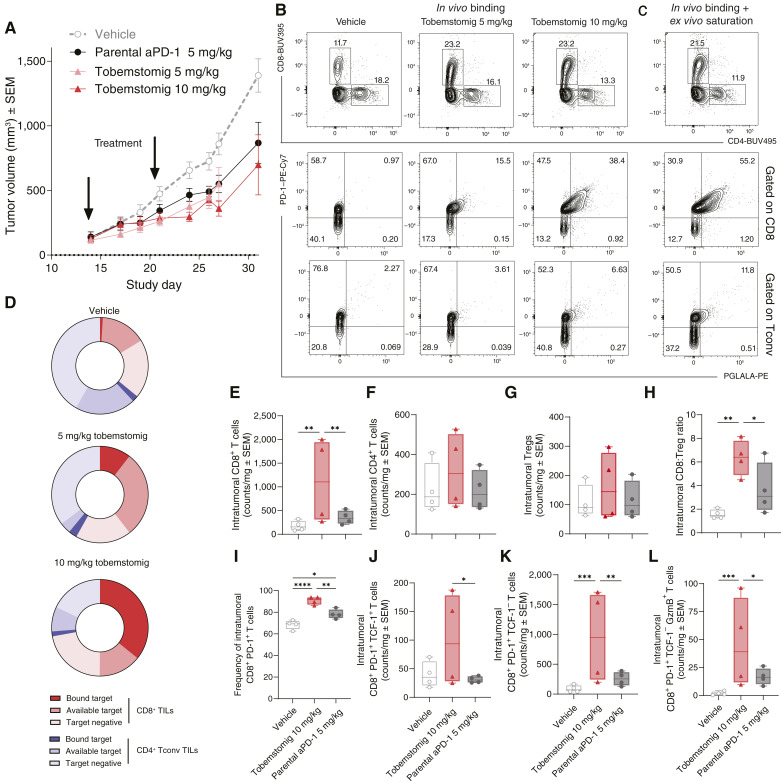
*In vivo* tobemstomig preferentially binds and expands CD8 TILs including PD-1^+^ TCF-1^+^ stem-like T cells and drives their differentiation toward cytotoxic effectors. *In vivo* efficacy study and immunopharmacodynamic in human PD-1 and human LAG-3 double transgenic mice bearing subcutaneous Panc02-H7-Fluc tumors treated for 2 weeks with the indicated treatments. **A,** Tumor growth curves (*n* = 12 mice per treatment group; mean ± SEM). **B** and **C,** Representative contour plots of frequencies of CD8 and CD4 TILs (top), PGLALA^+^ PD-1^+^ CD8^+^ TILs (middle), and PGLALA^+^ PD-1^+^ CD4^+^ TILs (bottom) in one mouse of four. **D,** Pie chart depicting dose-dependent binding of tobemstomig as PGLALA^+^, available target, and target-negative cells within CD8 and CD4 TILs. **E–L,** Immunopharmacodynamic analysis of the effect of tobemstomig vs. parental anti–PD-1 (aPD-1) on (**E**) CD8, (**F**) CD4 T cells, and (**G**) Treg counts within the tumor microenvironment, (**H**) CD8:Treg ratio, (**I**) frequencies of PD-1^+^ CD8 TILs, (**J**) amount of stem-like (PD-1^+^ TCF-1^+^) CD8 T cells, and (**K** and **L**) progeny (*n* = 4; box plots represent median, minimum/maximum, and individual points). Statistical comparisons were performed using one-way ANOVA with the Tukey multiple comparison test (*, *P* < 0.05; **, *P* < 0.01; ***, *P* < 0.001; ****, *P* < 0.0001).

The mice were treated once a week for only 2 weeks with either tobemstomig or parental anti–PD-1. On day 24, some of the mice were sacrificed for *in vivo* binding assessment and immunopharmacodynamic analysis, whereas the rest of the mice were further monitored for efficacy. Tobemstomig better controlled the tumor growth than parental anti–PD-1, demonstrating the contribution of the LAG-3 blockade in addition to PD-1 inhibition (Fig. 3A). *Ex vivo* staining of tumors isolated from treated mice with a PE-labeled antibody detecting the PGLALA mutation revealed that after two *in vivo* treatments, tobemstomig was found on the surface of CD8 TILs in a dose-dependent manner (Fig. 3B–D; Supplementary Fig. S3). In addition, both the doses of tobemstomig drove the *in vivo* selective expansion of target-positive CD8 TILs when compared with the vehicle group (Fig. 3D; Supplementary Fig. S3). Immunopharmacodynamic analysis highlighted a significant increase in the number of CD8 TILs upon tobemstomig treatment (Fig. 3E), with no significant changes in the total counts of CD4 TILs (Fig. 3F) and Tregs (Fig. 3G), resulting in a significantly higher CD8:Treg ratio than the other groups (Fig. 3H).

Interestingly, tobemstomig significantly increased the frequencies of PD-1^+^ CD8 TILs (Fig. 3I), a *bona fide* marker of tumor-specific T cells (20, 39–41), together with the increase in the number of PD-1^+^ TCF-1^+^ stem-like TILs (Fig. 3J) and their progeny (Fig. 3K). Within the PD-1^+^ TCF-1^−^ CD8 TILs, tobemstomig elicited the acquisition of a more cytotoxic effector profile than the vehicle and parental anti–PD-1 (Fig. 3L).

### Tobemstomig maintains long-term PD-1^+^ TCF-1^+^ CD8 T cells and their progeny in the tumor

We then used transgenic mice challenged subcutaneously with Panc02-H7-Fluc tumors to compare the efficacy of tobemstomig with nivolumab and relatlimab-like monotherapies, as well as their combination, and further investigated their mechanism of action. This time we used a murinized (mu) version of tobemstomig, nivolumab, and relatlimab-like anti–LAG-3 to prevent the formation of antidrug antibodies and to prolong the treatment to 3 weeks. Some mice were sacrificed either on day 28 or 37, and the remaining mice were further monitored for efficacy.

**Figure 4.**
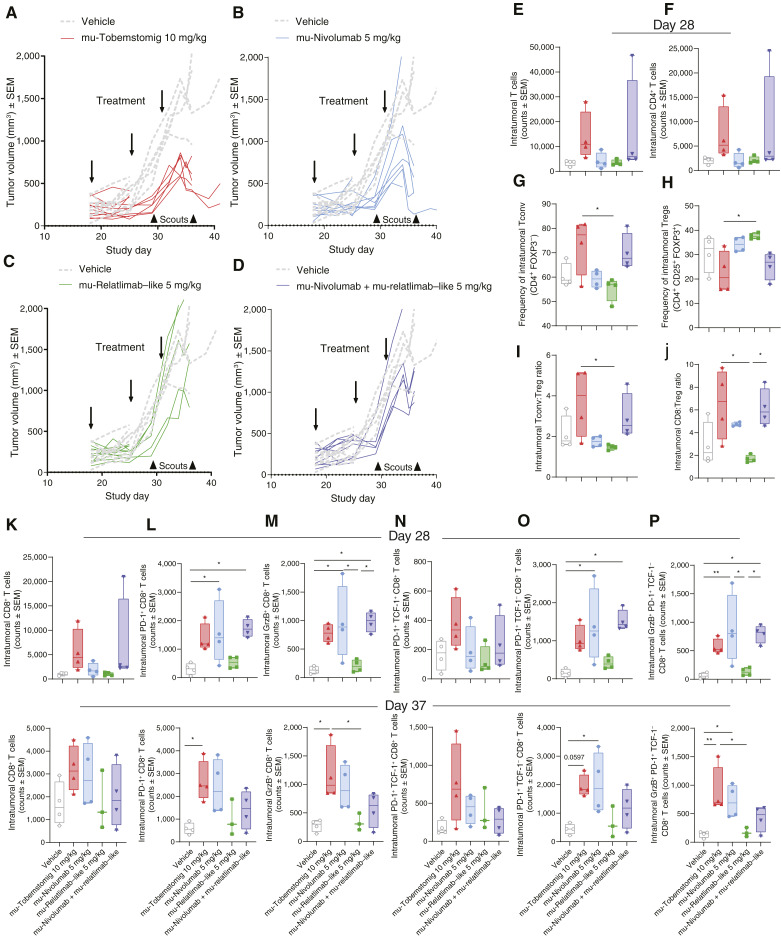
Tobemstomig maintains long-term PD-1^+^ TCF-1^+^ CD8 T cells and their progeny in the tumor. *In vivo* efficacy study and immunopharmacodynamic in human PD-1 and human LAG-3 double transgenic mice bearing subcutaneous Panc02-H7-Fluc tumors treated for 3 weeks with the indicated treatments. Tumor growth curves in mice receiving (**A**) mu-tobemstomig, (**B**) mu-nivolumab, (**C**) mu-relatlimab–like anti–LAG-3, and (**D**) mu-nivolumab in combination with mu-relatlimab–like anti–LAG-3 (*n* = 10 mice per treatment group, mean ± SEM). **E,** Amount of intratumoral T cells, (**F**) amount of intratumoral CD4 T cells, (**G**) frequency of Tconv cells (CD4^+^ FOXP3^−^), (**H**) frequency of Tregs (CD4^+^ FOXP3^+^), (**I**) Tconv:Treg ratio, (**J**) CD8:Treg ratio within the tumor of mice sacrificed on day 28, (**K**) amount of intratumoral CD8 T cells, (**L**) of which PD-1^+^, (**M**) granzyme B^+^ (GrzB^+^), (**N**) PD-1^+^ TCF-1^+^ stem-like, and (**O**) PD-1^+^ TCF-1^−^ progeny with (**P**) cytotoxic effector functions (*n* = 4; box plots represent median, minimum/maximum, and individual points). Statistical comparisons were performed using one-way ANOVA with the Tukey multiple comparison test (*, *P* < 0.05; **, *P* < 0.01).

We observed that all the mice receiving mu-tobemstomig had better tumor growth control than the other treatment groups (Fig. 4A). In contrast, treatment with mu-nivolumab monotherapy showed TGI in some of the treated mice (Fig. 4B), whereas most of the mice treated with mu-relatlimab–like anti–LAG-3 had a tumor growth similar to the vehicle group (Fig. 4C). The combination of mu-relatlimab–like with mu-nivolumab displayed in some mice an early control of the tumor growth followed by tumor escape in all treated mice (Fig. 4D). Immunopharmacodynamic analysis at day 28 revealed that both mu-tobemstomig and the combination of mu-nivolumab with mu-relatlimab–like increased the counts of intratumoral T cells (Fig. 4E), including CD4^+^ T cells (Fig. 4F), and significantly favored the increase in frequencies of Tconv cells (Fig. 4G) over Tregs (Fig. 4H), resulting in a higher Tconv:Treg ratio (Fig. 4I). Interestingly, mu-relatlimab–like anti–LAG-3 monotherapy showed the opposite trend by increasing the frequencies of Tregs over Tconv cells (Fig. 4G and H). Similarly, mu-tobemstomig and the combination of mu-nivolumab with mu-relatlimab–like resulted in a higher CD8:Treg ratio in the tumors (Fig. 4J) by expanding the counts of intratumoral CD8 T cells [Fig. 4K (top)]. Monotherapy with mu-relatlimab–like anti–LAG-3 did not change CD8 TIL:Treg ratio when compared with the untreated group given that it did not change the counts of CD8 TILs (Fig. 4J and K).

A deeper characterization of the phenotype of CD8 TILs at day 28 highlighted that mu-nivolumab alone and in combination with mu-relatlimab–like anti–LAG-3, besides increasing the counts of CD8 TILs [Fig. 4K (top)], significantly expanded the PD-1^+^ tumor–specific [Fig. 4L (top)] and cytotoxic ones [Fig. 4M (top)]. In addition, the combination of mu-relatlimab–like anti–LAG-3 with mu-nivolumab, without changing the amounts of stem-like CD8 T cells in the tumor [Fig. 4N (top)], significantly drove the expansion and differentiation of a functional progeny producing Granzyme B [Fig. 4O and P (top); Supplementary Fig. S4A–S4C]. However, by day 37, corresponding with the escape of the tumor growth, the phenotype of the CD8 TILs isolated from the tumors of mice treated with mu-relatlimab–like anti–LAG-3 in combination with mu-nivolumab did not significantly differ from the one found in the control group [Fig. 4K–P (bottom); Supplementary Fig. S4A–S4C]. Strikingly, mu-tobemstomig, while showing similar trends to the combination of mu-nivolumab with mu-relatlimab–like at day 28, by day 37 elicited further expansion of CD8 TILs [Fig. 4K (bottom)], which were mainly PD-1^+^ [Fig. 4L (bottom)] and endowed with cytotoxic effector functions [Fig. 4M (bottom)]. Notably, mu-tobemstomig showed the ability to expand stem-like T cells at both time points [Fig. 4N (top and bottom); Supplementary Fig. S4A–S4C], while sustaining their differentiation toward cytotoxic effectors [Fig. 4O and P (bottom); Supplementary Fig. S4A–S4C], which correlated with a better control of the tumor growth (Fig. 4A).

**Figure 5.**
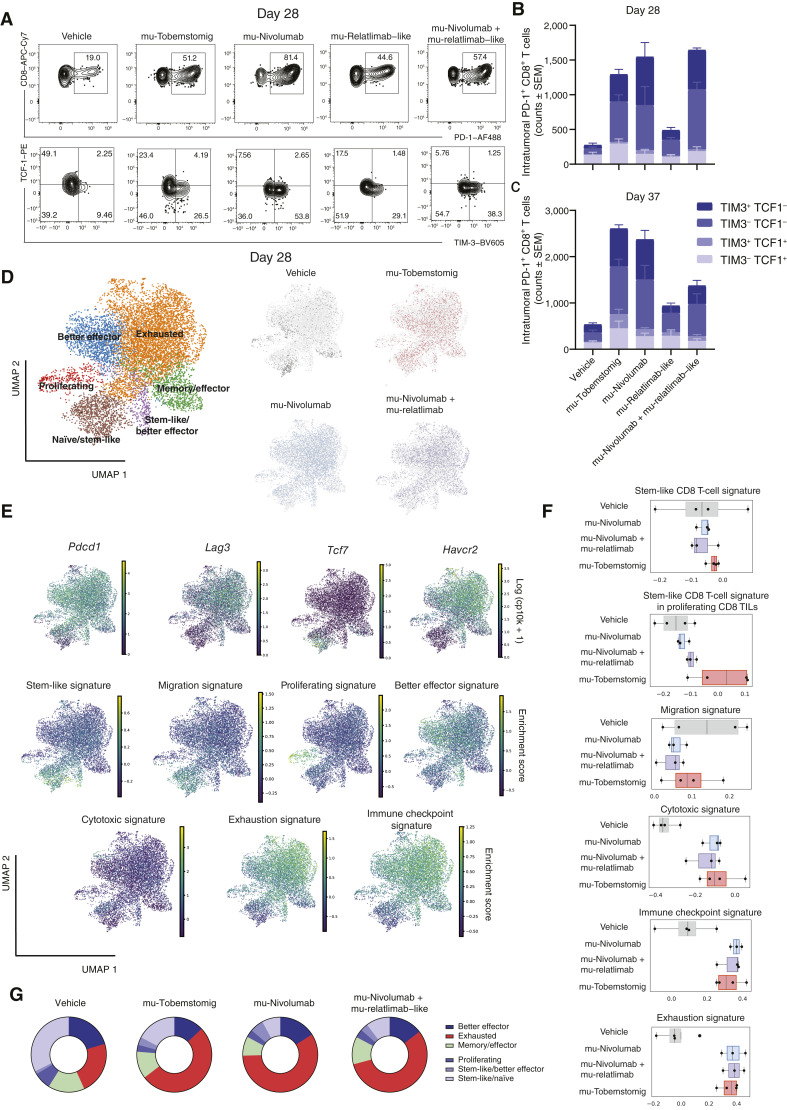
Intratumoral PD-1^+^ TCF-1^+^ stem-like CD8 T cells retain their transcriptional characteristics upon treatment with mu-tobemstomig. Immunopharmacodynamic study on the effect of different therapies, given three times, on amounts, phenotype, effector function, and molecular signature of intratumoral CD8 T cells obtained from human PD-1 and human LAG-3 double transgenic mice bearing subcutaneous Panc02-H7-Fluc tumors. **A,** Representative contour plots of the frequencies of PD-1^+^ CD8 TILs expressing TCF-1 (stem-like) vs. TIM-3 (progeny). Amounts of intratumoral PD-1^+^ CD8 TILs expressing TCF-1 vs. TIM-3 (**B**) at day 28 and (**C**) day 37 (*n* = 4, stacked bars; mean ± SEM). **D,** Two-dimensional Uniform Manifold Approximation and Projection for Dimension Reduction (UMAP) visualization of CD8^+^ TILs colored according to the subset (left) and specific treatment effect (right). **E,** Expression of selected markers and signatures among CD8^+^ TILs using a 2D UMAP visualization. **F,** Average signature enrichment scores among CD8 T cells per treatment group and individual animal (3–4 mice per group; box plots represent median, minimum/maximum, and individual points). **G,** Pie chart represents relative cell-type composition per treatment group.

To better understand the differentiating mechanism of action of tobemstomig from the combination of nivolumab and relatlimab-like anti–LAG-3, we focused on the expression of TCF-1 and TIM-3 within the PD-1^+^ CD8^+^ TILs to discriminate between stem-like and exhausted T cells. Mu-Tobemstomig maintained (Fig. 5A and B) and expanded the intratumoral reservoir of PD-1^+^ TCF-1^+^ stem-like T cells by day 37 (Fig. 5C) and increased the amounts of CD8 TILs with intermediate differentiation states (Fig. 5B and C; Supplementary Fig. S5A–S5C), suggesting a more sustained antitumor response (42) and better efficacy. Conversely, mu-nivolumab monotherapy and its combination with mu-relatlimab–like anti–LAG-3 elicited a decrease in the frequencies and amounts of stem-like T cells in favor of more differentiated and exhausted phenotypes (Fig. 5A–C; Supplementary Fig. S5A–S5C), in line with recent reports (43).

To corroborate our findings, we performed scRNA-seq on the same tumor samples collected on day 28. We found both qualitative and quantitative differences between vehicle, mu-tobemstomig, mu-nivolumab monotherapy, and in combination with mu-relatlimab–like anti–LAG-3 (Fig. 5D–G; Supplementary Fig. S5D and S5E). The CD8 TILs generated by mu-tobemstomig possessed a higher stem-like, proliferating stem–like, and migration signature score, indicating that they retained some of the transcriptional characteristics of the stem-like PD-1^+^ TCF-1^+^ CD8 TILs (Fig. 5F; Supplementary Fig. S5D–S5F). In addition, their progeny displayed a trend for higher cytotoxic signature (Fig. 5F). In contrast, the immune checkpoint and exhaustion signature scores were higher in CD8 TILs generated by mu-nivolumab alone and in combination with mu-relatlimab–like anti–LAG-3 (Fig. 5F; Supplementary Fig. S5F).

Relative cell-type composition per treatment group confirmed that mu-tobemstomig was able to better maintain the population of stem-like CD8 TILs (Fig. 5G), whereas mu-nivolumab alone and in combination with mu-relatlimab–like anti–LAG-3 eroded the stem-like pool in favor of exhausted CD8 TILs (Fig. 5G).

Altogether, these data indicate that tobemstomig provides deeper and more durable efficacy than nivolumab in combination with relatlimab-like anti–LAG-3 by sustaining overtime the amounts of tumor-specific TILs, including stem-like T cells and their functional progeny, at the expense of Tregs.

## Discussion

Despite the unprecedented efficacy of immune CPIs targeting PD-(L)1 and CTLA-4 for the treatment of several malignancies, many patients with cancer do not benefit from such treatments because of primary or secondary resistance mechanisms, tumor-immune contexture, and Tregs (5).

One potential reason for the development of resistance toward anti–PD-1 therapy is the coexpression of additional immune checkpoints with nonredundant regulatory functions like LAG-3, TIM-3, and TIGIT by tumor-specific T cells (6–13).

LAG-3, the first discovered immune checkpoint (6), is expressed by CD8 and CD4 TILs. Both PD-1 and LAG-3 are established markers of T-cell exhaustion (8, 14–18) in cancer and chronic viral infections; however, it has been recently reported that both receptors are also expressed by stem-like T cells (9, 19, 20), a population critical for the response to anti–PD-1 therapy (15, 19, 21, 22). On stem-like T cells, both PD-1 and LAG-3 receptors prevent their expansion, differentiation, and acquisition of effector functions (9, 11) against the tumor, therefore, providing the rationale for therapeutically blocking both PD-1 and LAG-3 pathways simultaneously.

There is strong preclinical (11, 12, 17, 23–25) and clinical (13, 26–28) evidence supporting the combination of antagonist therapies targeting both the PD-1 and LAG-3 receptors, including the results of a phase II/III clinical trial in first-line melanoma by Bristol Meyer Squibb, showing clinical benefit of the combination of nivolumab and relatlimab over nivolumab alone (26). This finding validates both the clinical relevance of the LAG-3 pathway in melanoma and the rationale for therapeutically cotargeting PD-1 and LAG-3 in clinical settings.

However, an important limitation to the efficacy of blocking the LAG-3 pathway in cancer might be the constitutive expression of LAG-3 by Tregs because LAG-3 limits Treg proliferation and suppressive function (29). Several independent lines of clinical and preclinical evidence support the concern that LAG-3 blockade may promote Treg expansion. Zhang and colleagues (29) demonstrated that LAG-3 deficiency specifically on Tregs enhanced their proliferation and suppressive function. Consistent with this, Huuhtanen and colleagues (28) reported that LAG-3^+^CD4^+^ T cells, including Tregs, which showed the highest LAG-3 expression within the CD4^+^ compartment, were the most prominently expanded population following nivolumab plus relatlimab treatment in patients with melanoma. Similarly, Lipson and colleagues (44) reported that nivolumab plus relatlimab significantly modulated four Treg subsets, with more CD4^+^ populations affected than CD8^+^ or NK populations.

These findings suggest that LAG-3 blockade with monospecific antibodies may carry an inherent risk of promoting Treg-mediated immunosuppression, potentially limiting their therapeutic benefit.

In this study, we describe tobemstomig, a novel heterodimeric BsAb to preferentially block PD-1 and LAG-3 on tumor-specific TILs by targeting both receptors on the same T cell through an avidity-driven selectivity gain. The monovalent binding to LAG-3 together with the higher affinity monovalent binding to PD-1 anchors tobemstomig on the T-cell surface preventing its internalization. This indicates a reduced sink effect and, therefore, a potentially improved bioavailability for tobemstomig over other bispecific formats and bivalent anti–LAG-3 antibodies. In addition, the silent-Fc portion of tobemstomig prevents drug-shaving by macrophages in the tumor microenvironment (37), a known adaptive resistance mechanism for IgG1- and IgG4-based therapies, potentially providing tobemstomig with an improved tumor retention and a higher receptor occupancy on TILs over time.

In an *in vitro* binding competition, tobemstomig showed to preferentially bind to conventional CD4 T cells over Tregs. The different expression levels of PD-1 and LAG-3 on the conventional CD4 T cells versus Tregs coupled with the higher affinity for PD-1 over LAG-3 are critical for the preferential targeting of tobemstomig. This translates in T-cell activation and acquisition of effector functions even in the presence of suppressive Tregs because of a reduced detrimental targeting of LAG-3 on Tregs.

*In vivo*, tobemstomig confirmed to preferentially target CD8 and conventional CD4 TILs over Tregs and to increase the frequencies of cytotoxic CD8 and CD4 TILs while decreasing those of Tregs in the tumor. As a result, tobemstomig demonstrated an improved TGI in several mouse tumor models, which was not recapitulated by the combination of anti–PD-1 and anti–LAG-3 antibodies. Importantly, in line with a previous report (28, 29, 44) and our *in vitro* findings, we observed that *in vivo* treatment with anti–LAG-3 monotherapy drove the expansion of Tregs over conventional CD4 and CD8 TILs and ultimately failed to provide control of the tumor growth. This observation provides concrete evidence supporting the development of a BsAb like tobemstomig with a reduced targeting of Tregs.

In the last couple of years, PD-1^+^TCF-1^+^ CD8 stem-like T cells have emerged as a key population in the immune response to chronic infections and cancer and to be critical for the success of CITs blocking PD-1/PD-L1 (1–3) and CTLA-4 (42). We have observed that tobemstomig maintains and expands PD-1^+^ TCF-1^+^ stem-like CD8 TILs and their functional progeny within tumor-specific CD8 TILs (20, 39–41) at detriment of intratumoral Tregs. Conversely, the combination of monospecific anti–PD-1 with anti–LAG-3 antibodies, like nivolumab with relatlimab-like, displayed a transitory beneficial expansion of PD-1^+^ tumor–specific CD8 TILs endowed with cytotoxic effector functions (13). This effect, however, was lost over time coinciding with the acquisition of an exhausted phenotype (13, 43), leading to tumor escape. The reduction in CD8 TILs observed in mice treated with the combination of nivolumab and relatlimab-like anti–LAG-3 could be the product of both the erosion over time of the pool of stem-like CD8 T cells (10, 43) and the detrimental expansion of Tregs upon LAG-3 blockade (28, 29, 44). Therefore, our observation on the effect of tobemstomig on replenishing the reservoir of stem-like CD8 T cells is particularly important in the aim to provide durable responses to patients with cancer.

Our preclinical observations have been validated in several clinical trials assessing the efficacy of tobemstomig in solid tumors (NCT04140500) and in several lines of treatment in melanoma (NCT04140500, NCT05116202, and NCT05419388; refs. 45, 46), including in Garralda and colleagues (45) and Long and colleagues (46), demonstrating the efficacy and mechanism of action of tobemstomig in CPI-experienced melanoma and neoadjuvant melanoma, respectively.

Long and colleagues (46) demonstrated that tobemstomig selectively engages PD-1/LAG-3 coexpressing TILs over LAG-3^+^ Treg cells, inducing robust immune activation and deep pathologic responses in stage III melanoma. Furthermore, Garralda and colleagues (45) reported that tobemstomig does not induce Treg expansion in peripheral blood or tumor, while enhancing CD8^+^ T-cell antitumor responses. This study presented the first proof of mechanism for a T-cell bispecific PD-1/LAG-3 dual CPI in CPI-experienced melanoma, based on the increases in CD8^+^ T cells and their cytotoxic effector functions, expansion of stem-like CD8^+^ T cells, and limited Treg expansion, validating the preclinical observations described in this article.

There is strong preclinical and clinical evidence supporting the benefit of coblocking PD-1 and LAG-3 over PD-1 alone. Nevertheless, an important limitation in achieving the full efficacy potential of LAG-3 blockade in cancer might come from the constitutive expression of LAG-3 on Tregs. LAG-3 blockade with anti–LAG-3 therapy might limit the overall efficacy by promoting the detrimental expansion of Treg, ultimately leading to the suppression of tumor-specific CD8 T-cell responses. We show here that tobemstomig, a BsAb, simultaneously blocks PD-1 and LAG-3 and preferentially targets CD8 TILs while reducing intratumoral Tregs. Tobemstomig specifically expands tumor-specific stem-like CD8 TILs and differentiates them into better effectors, resulting in superior, durable tumor inhibition in several mouse tumor models. Conversely, the combination of monospecific antibodies targeting PD-1 and LAG-3 separately failed to recapitulate the efficacy observed for tobemstomig and eroded the stem-like T-cell pool over time, leading CD8 TILs to acquire an exhausted phenotype while eliciting the detrimental expansion of Tregs in the tumor.

## Supplementary Material

Supplementary Figure 1 Tobemstomig is refractory to drug-shaving by macrophages

Supplementary Figure 2 In-vivo dose dependent preferential binding of tobemstomig

Supplementary Figure 3 In-vivo and ex-vivo preferential binding of tobemstomig

Supplementary Figure 4 Tobemstomig maintains long-term stem-like T cells and their progeny

Supplementary Figure 5 Intratumoral stem-like T cells retain their profile upon tobemstomig treatment

## Data Availability

The scRNA-seq data discussed in this publication have been deposited in ArrayExpress with the accession number E-MTAB-15144. Other data generated in this study are available upon request to the corresponding author.

## Code Availability

For the scRNA-seq data analysis, we used Cell Ranger versions 6.0.0 and 8.0.1; Scanpy versions 1.6.0 and 1.10.0; Besca versions 2.4 and 2.5.3; and Scirpy version 0.7.1; all additional custom workbooks are available at https://github.com/bedapub/tobemstomig_in_vivo_TILs_Panc02_publication.
