# Supplementary material for: Tobemstomig, a Novel Bispecific Antibody, Preferentially Blocks PD-1 and LAG-3 on CD8 TILs to Expand Stem-like T Cells for Sustained Tumor Control
Source: Cancer Res Commun. 2026 Jul 9;6(7):1619–39. doi: 10.1158/2767-9764.CRC-26-0207 (PMC13347385; doi:10.1158/2767-9764.CRC-26-0207)
Supplement: Supplementary Figure 1 — Tobemstomig is refractory to drug-shaving by macrophages [file crc-26-0207_supplementary_figure_1_suppsf1.pdf]

Supplementary Fig. 1

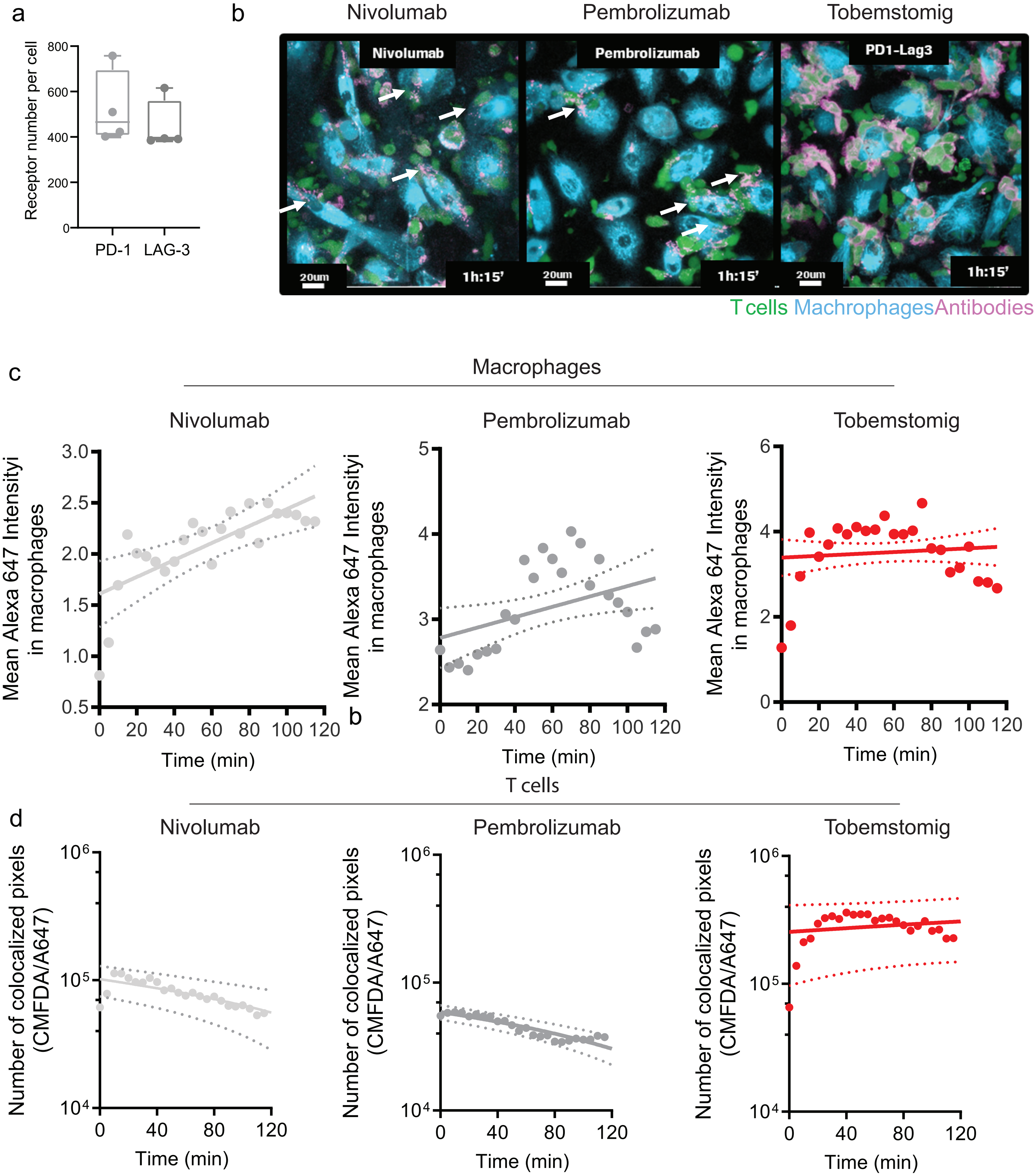

Supplementary Figure 1. Tobemstomig is refractory to drug-shaving by macrophages

a. Amount of PD-1and LAG- receptors per T cell on in-vitro polyclonally activated CD4 T cells (n=4 healthy donors, 2 independent experiments; box plots representing median, minimum/maximum and individual points). **b.** Representative imaging at 1hour and 15 minutes of macrophages (light blue) cultured with activated CD8 T cells (green) pre-stained with either AF-647 labelled parental anti-PD-1, nivolumab, pembrolizumab or tobemstomig. White arrows show the location in which the shaving is taking place. **c.** Intensity data of the fluorescent antibodies from IMARIS plotted over time with a linear regression of 95% prediction for each treatment. **d.** Normalized dataset over control parental anti-PD-1 to define the percentage of loss of signal from the T cells as drug-shaving for each treatment. A linear regression was then calculated based on normalized data.
