# Supplementary material for: Tobemstomig, a Novel Bispecific Antibody, Preferentially Blocks PD-1 and LAG-3 on CD8 TILs to Expand Stem-like T Cells for Sustained Tumor Control
Source: Cancer Res Commun. 2026 Jul 9;6(7):1619–39. doi: 10.1158/2767-9764.CRC-26-0207 (PMC13347385; doi:10.1158/2767-9764.CRC-26-0207)
Supplement: Supplementary Figure 2 — In-vivo dose dependent preferential binding of tobemstomig [file crc-26-0207_supplementary_figure_2_suppsf2.pdf]

Supplementary Fig. 2

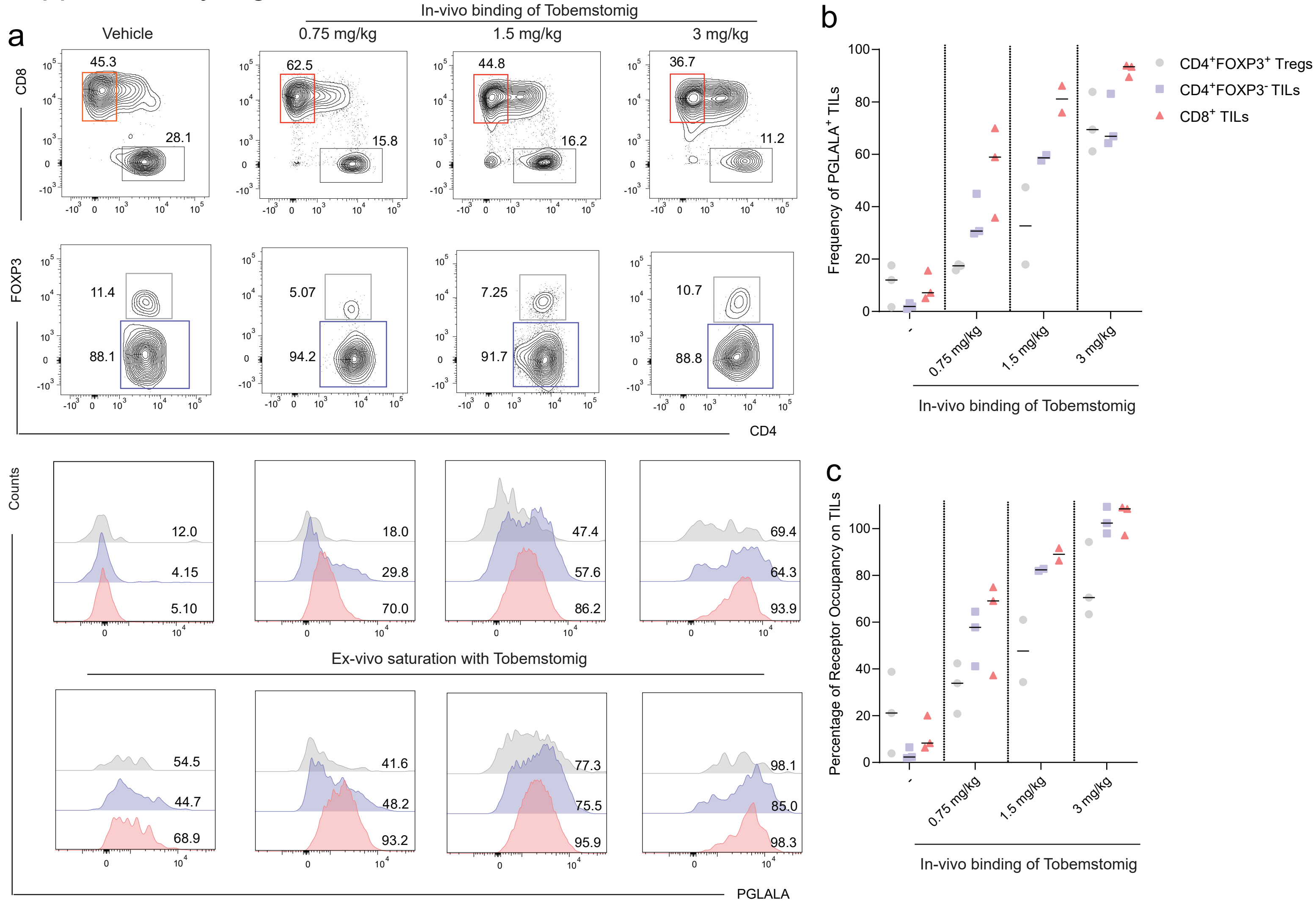

Supplementary Figure 2. In-vivo dose dependent preferential binding of tobemstomig to CD8 TILs over Tconv and Tregs in OCI-Ly18 tumors

In-vivo binding study of tobemstomig to CD8 and CD4 TILs and Tregs isolated from OCI-Ly18 tumors obtained from $CD34^+$ NSG humanized mice treated with either 0.75, 1.5 or 3 mg/kg of tobemstomig. **a.** Representative contour plots depicting frequencies of CD8 and CD4 TILs (top) and Tregs ($CD4^+$ $FOXP3^+$) (middle). Representative histogram plots of in-vivo bound tobemstomig (middle) versus ex-vivo saturated samples (bottom) across all three doses of tobemstomig on CD8 TILs (red), CD4 TILs (blue) and Tregs (grey). **b.** Frequency of $PGLALA^+$ TILs indicating in-vivo binding of tobemstomig at the indicated doses. **c.** Receptor occupancy of tobemstomig on CD8 TILs and Tconv, and Tregs across doses (n=3 mice per treatment group, median and individual points).
