# Supplementary material for: Tobemstomig, a Novel Bispecific Antibody, Preferentially Blocks PD-1 and LAG-3 on CD8 TILs to Expand Stem-like T Cells for Sustained Tumor Control
Source: Cancer Res Commun. 2026 Jul 9;6(7):1619–39. doi: 10.1158/2767-9764.CRC-26-0207 (PMC13347385; doi:10.1158/2767-9764.CRC-26-0207)
Supplement: Supplementary Figure 3 — In-vivo and ex-vivo preferential binding of tobemstomig [file crc-26-0207_supplementary_figure_3_suppsf3.pdf]

Supplementary Fig. 3

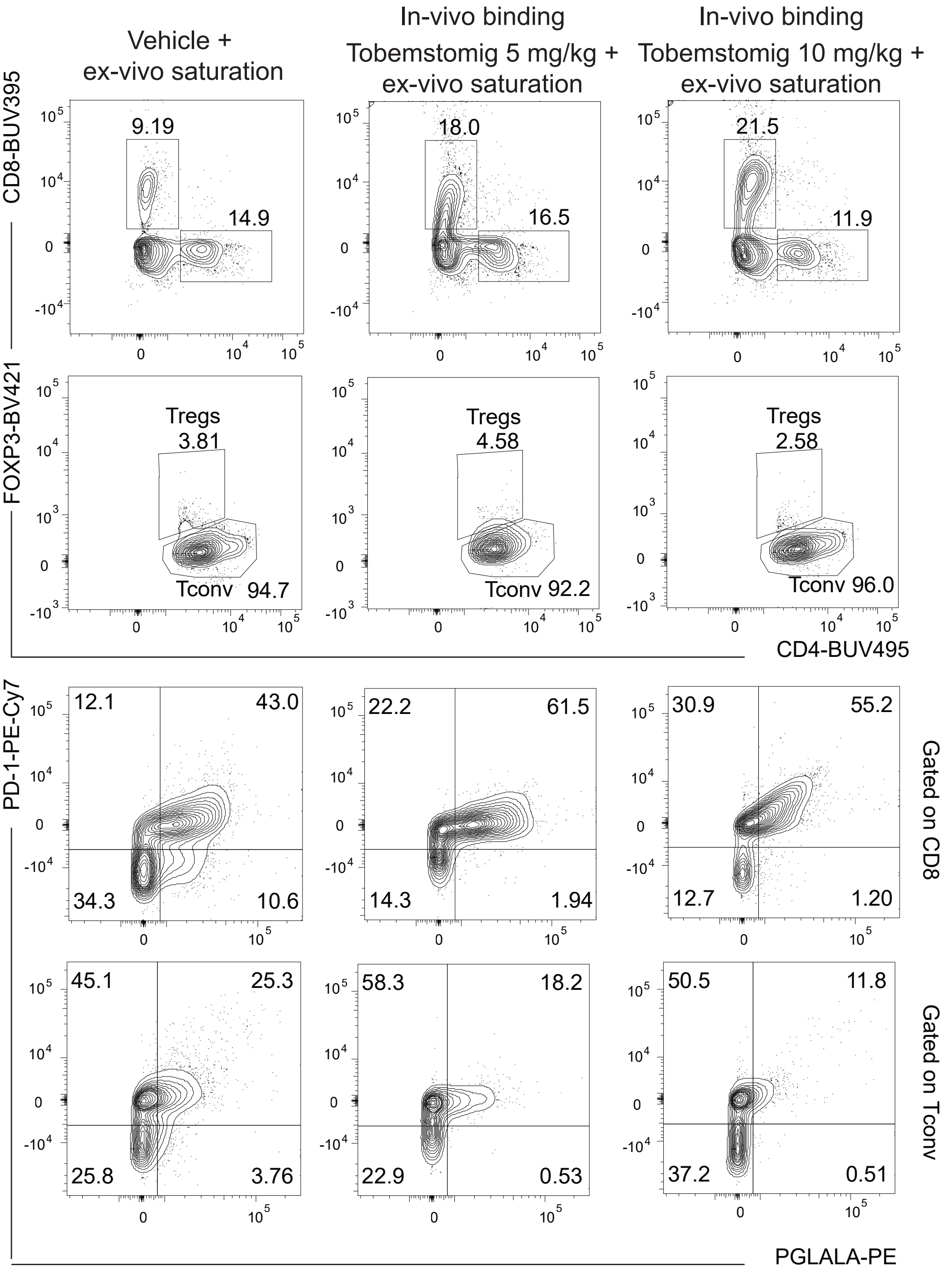

Supplementary Fig. 3. In-vivo and ex-vivo preferential binding of tobemstomig to CD8 TILs over Tconv in Panc02-H7-Fluc tumors

In-vivo binding study of tobemstomig to CD8 and CD4 TILs and Tregs isolated from Panc02-H7-Fluc tumors obtained from immunocompetent human PD-1, human LAG-3 double transgenic mice treated with either 5 or 10 mg/kg of tobemstomig. Representative contour plots depicting frequencies of CD8 and CD4 TILs (top) and Tregs ($CD4^{+}$ $FOXP3^{+}$) (middle) and PD-1^{+} $PGLALA^{+}$ CD8 (middle) and CD4 TILs (bottom) in one mouse out of four upon two in-vivo treatments with tobemstomig and ex-vivo saturation with tobemstomig.
