# Supplementary material for: Tobemstomig, a Novel Bispecific Antibody, Preferentially Blocks PD-1 and LAG-3 on CD8 TILs to Expand Stem-like T Cells for Sustained Tumor Control
Source: Cancer Res Commun. 2026 Jul 9;6(7):1619–39. doi: 10.1158/2767-9764.CRC-26-0207 (PMC13347385; doi:10.1158/2767-9764.CRC-26-0207)
Supplement: Supplementary Figure 4 — Tobemstomig maintains long-term stem-like T cells and their progeny [file crc-26-0207_supplementary_figure_4_suppsf4.pdf]

Supplementary Fig. 4

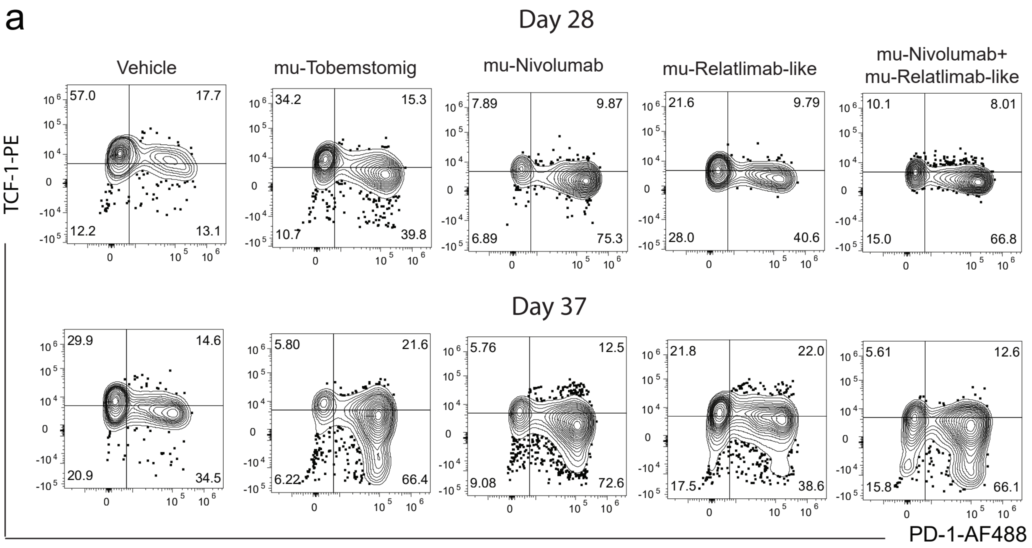

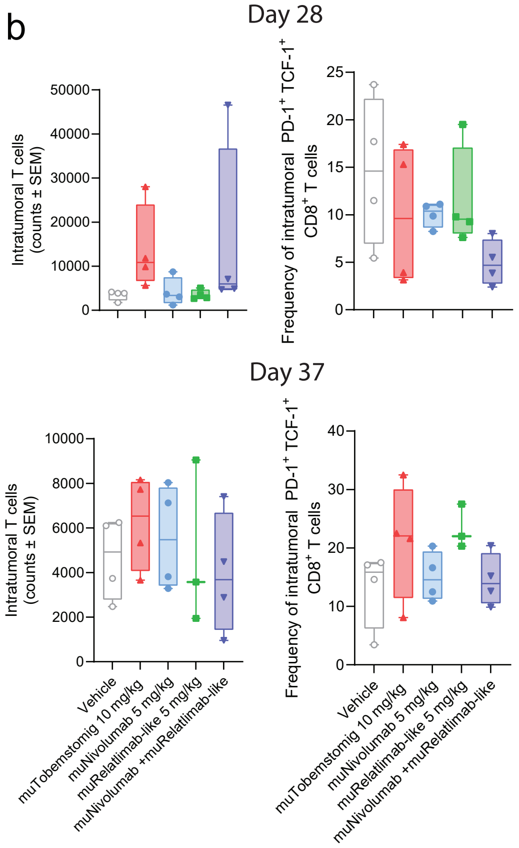

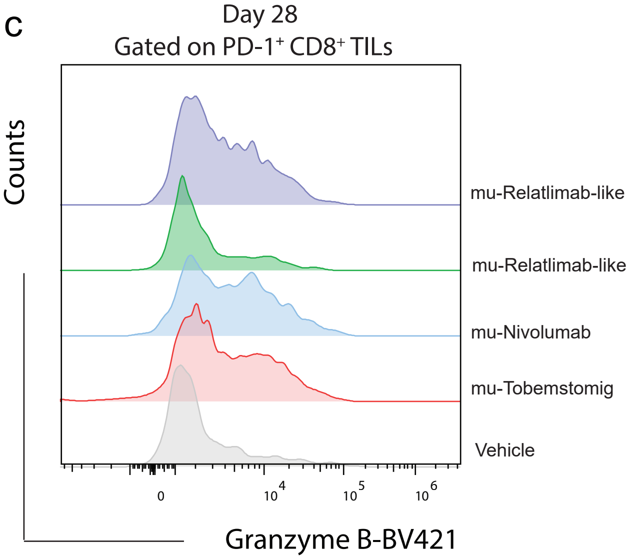

Supplementary Fig. 4. Tobemstomig maintains long-term PD-1$^+$ TCF-1$^+$ CD8 T cells and their progeny in the tumor

Immuno-pharmacodynamic in human PD-1, human LAG-3 double transgenic mice bearing subcutaneous Panc02-H7-Fluc tumors treated for 3 weeks with the indicated treatments. **a.** Representative contour plots depicting within CD8 TILs PD-1$^+$ TCF-1$^+$ stem-like and PD-1$^+$ TCF-1$^-$ progeny on day 28 and 37 of one individual mouse out of four. **b.** Amount of intratumoral T cells and frequencies of PD-1$^+$ TCF-1$^+$ CD8 TILs at day 28 (top) and 37 (bottom). **c.** Representative histogram plot of granzyme B within PD-1$^+$ CD8 TILs of one mouse per treatment out of four each.
