# Supplementary material for: Tobemstomig, a Novel Bispecific Antibody, Preferentially Blocks PD-1 and LAG-3 on CD8 TILs to Expand Stem-like T Cells for Sustained Tumor Control
Source: Cancer Res Commun. 2026 Jul 9;6(7):1619–39. doi: 10.1158/2767-9764.CRC-26-0207 (PMC13347385; doi:10.1158/2767-9764.CRC-26-0207)
Supplement: Supplementary Figure 5 — Intratumoral stem-like T cells retain their profile upon tobemstomig treatment [file crc-26-0207_supplementary_figure_5_suppsf5.pdf]

Supplementary Fig. 5

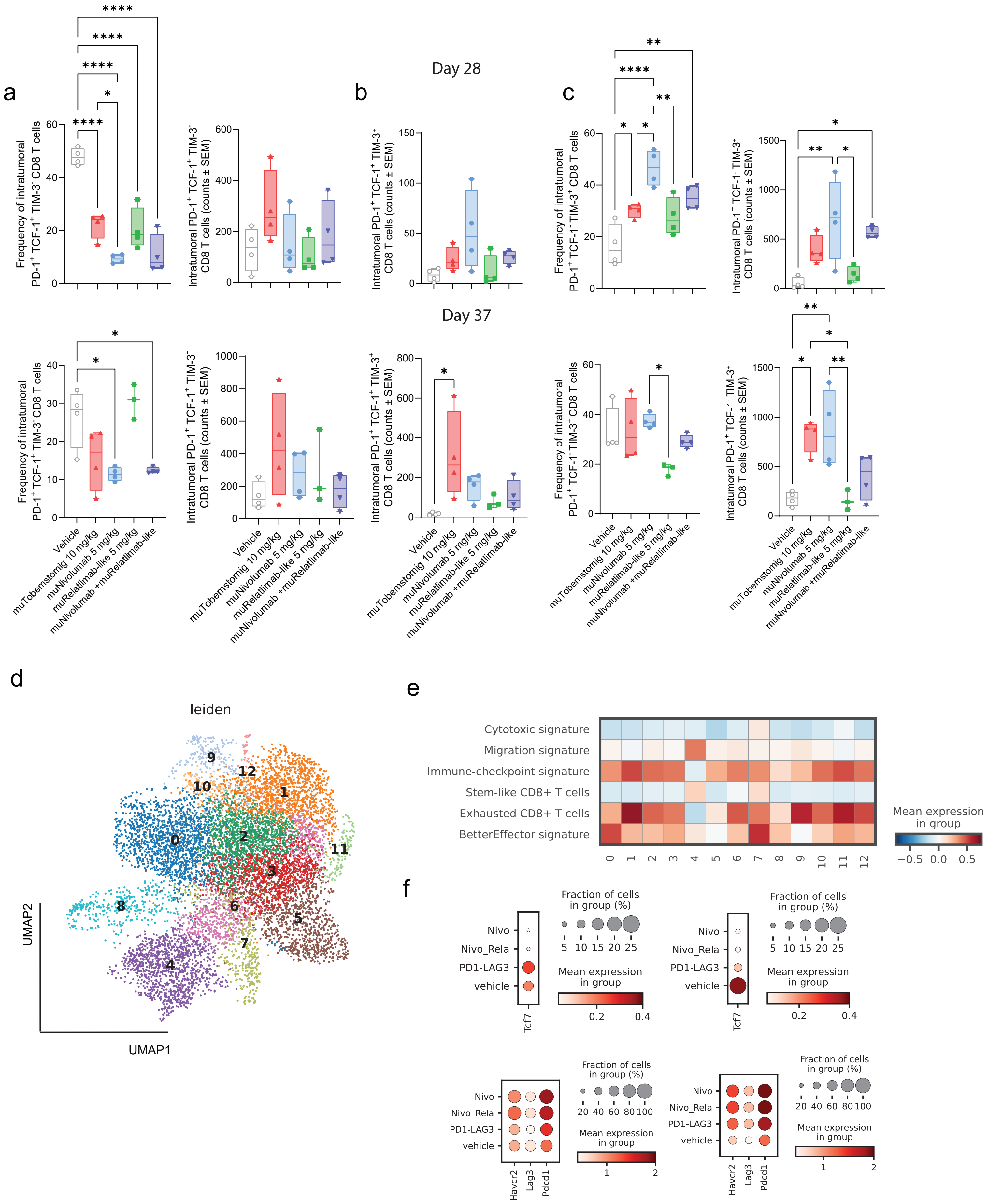

Supplementary Fig. 5. Intratumoral PD-1^{+} TCF-1^{+} stem-like CD8 T cells retain their transcriptional characteristics upon treatment with mu-tobemstomig

Immuno-pharmacodynamic study on the effect of the different therapies, given three times, on amounts, phenotype, effector function and molecular signature of intra-tumoral CD8 T cells obtained from human PD-1, human LAG-3 double transgenic mice bearing subcutaneous Panc02-H7-Fluc tumors. **a.** Frequencies and amounts of PD-1^{+} CD8 TILs expressing TCF-1^{+} TIM-3^{-}(stem-like), **b.** TCF-1^{+} TIM-3^{+} (intermediate) versus **c.** TCF-1^{-} TIM-3^{+} (progeny) (n=4 mice per treatment group, box plots representing median, minimum/maximum and individual points). **d.** Joint 2D UMAP visualization of all cells across all treatments and individual mice colored according to Leiden clusters. **e.** Average relative expression of selected genes across the distinct T cell subsets within the CD8^{+} TILs depicted in Fig. 5d. **f.** Average expression of TCF-1 (Tcf7), TIM-3 (Havcr2), LAG-3 (Lag3), and PD-1 (Pdcd1) per treatment condition in proliferating CD8+ T cells (left panels) and other CD8+ T cells (right panels). The size of the circles indicates the fraction of cells expressing the respective gene in each condition (3-4 mice per group). Statistical comparisons were performed using one-way ANOVA with Tukey's multiple comparison test. (* = p<0.05; **=p <0.01, ***= p <0.001, ****= p <0.0001).
